# Ferroptosis is a novel pathogenic mechanism of FDXR-related disease via disruption of the NRF2 pathway

**DOI:** 10.1038/s41420-025-02840-y

**Published:** 2025-12-23

**Authors:** Teresa Campbell, Jesse Slone, Jimmy Vu, Wensheng Liu, Li Yang, Adam Dourson, Luis F. Queme, Michael P. Jankowski, Taosheng Huang

**Affiliations:** 1 Department of Pediatrics, Jacobs School of Medicine and Biomedical Sciences, University at Buffalo, Buffalo, NY USA; 2 Division of Human Genetics, Cincinnati Children’s Hospital Medical Center, Cincinnati, OH USA; 3 College of Medicine, University of Cincinnati, Cincinnati, OH USA; 4 Department of Pediatrics, Xiangya Hospital; Central South University, Changsha, China; 5 Department of Anesthesia, Division of Pain Management, Cincinnati Children’s Hospital Medical Center, Cincinnati, OH USA; 6 Department of Biomedical Sciences, University of New England College of Osteopathic Medicine, Biddeford, ME USA; 7 Present Address: Institute of Medical Genetics & Genomics, Fudan University, 1st Floor, Building B, Suite 1027, Research Building 2, No. 131 Dong’an Road, Xuhui District Shanghai, China

**Keywords:** Cell death, Neurodegenerative diseases

## Abstract

Loss-of-function variants in the ferredoxin reductase *(FDXR)* gene result in a primary mitochondrial disease in humans, involving abnormal mitochondrial iron accumulation. However, the molecular mechanism is not fully understood. To better understand the underlying pathology of FDXR-related disease, we generated a mouse model corresponding to the hotspot variant found in humans. We demonstrated increased lipid peroxidation in the inner mitochondrial and plasma membranes, resulting in susceptibility to ferroptosis. Closer examination revealed that disruption of the NRF2 pathway and its target gene *SLC7A11* appear to play important roles in this pathogenic process. Finally, administration of the NRF2 activator *omaveloxolone*, which was recently approved by the FDA for treatment of Friedreich’s ataxia, helps mitigate the pathogenesis. Together, our results suggest that ferroptosis is a novel underlying mechanism of FDXR-related disease and that activation of NRF2 could be an immediate, viable treatment option for individuals with FDXR-related disease and other conditions involving aberrant iron metabolism.

## Introduction

Iron is an important mediator of electron transfer in cellular redox reactions. A significant portion of cellular iron is utilized in the mitochondria [1], an organelle traditionally associated with the generation of ATP and bioenergetic disorders [2]. Within the mitochondria, iron has two very critical functions: iron-sulfur cluster (ISC) biogenesis and heme synthesis. In particular, pathogenic variants in the genes encoding enzymes required for ISC biogenesis have been linked to a variety of rare neurodegenerative and metabolic disorders, with many presenting in early childhood [3–8]. Moreover, iron overload in the mitochondria has been documented in disorders of heme or ISC metabolism, notably Friedreich’s ataxia (FRDA) [9, 10]. Mitochondrial iron overload is a significant pathology, as accumulation corresponds to reactive oxygen species (ROS) accrual, possibly triggering cell death via elevated production of the deleterious hydroxyl radical from Haber-Weiss and Fenton reaction products [11].

Ferroptosis is an iron-dependent form of programmed cell death caused by redox dysregulation [12]. While the progression of ferroptosis involves multiple cellular pathways, frequent hallmark features include the accumulation of lipid peroxidases in the cellular membrane and depletion of the antioxidant system based around glutathione (GSH), the cystine/glutamate antiporter “system X_c_^-^” (consisting of the proteins SLC7A11 and SLC3A2), and glutathione peroxidase 4 (GPX4) [13, 14]. Interestingly, despite the name, the role of iron in ferroptosis has yet to be fully elucidated, although iron accumulation and an excess labile iron pool are often observed in cells undergoing ferroptosis [13]. Overall, recent literature has classified ferroptosis inducer molecules (or “FINs”) into 4 classes on the basis of their targets and cellular mechanisms: class I FINs act by depleting the system Xc-/GSH pathway; class II FINs directly target or block GPX4; class III FINs degrade both GPX4 and CoQ_10_; and class IV FINs increase the labile iron pool [15–17]. Thus, different ferroptosis inhibitors as well as different forms of genetic disease involving ferroptosis are likely to work through distinct cellular mechanism, and could respond to different therapeutic strategies. For example, iron chelators such as DFO would be expected to work well in preventing FINs or genetic disorders operating through the class IV mechanism (i.e. increased labile iron in the cell), but might be less ideal for other classes of FINs.

Over the last decade, ferroptosis has been implicated in multiple disease states, including Alzheimer’s disease, cardiovascular disease, infertility, and various neurodegenerative disorders [18–23]. Moreover, ferroptosis has been implicated in the pathology of FRDA and other iron metabolic disorders [19]. For this reason, the identification of cellular pathways which combat ferroptosis are of great interest for pharmaceutical development. Nuclear factor erythroid 2-related factor 2 (NRF2) is a transcription factor which initiates during times of oxidative stress. During homeostasis, NRF2 is held in the cytoplasm by KEAP1 (Kelch-like ECH-associated protein 1), an adapter of the E3-ubiquitin ligase, CUL3 [24]. KEAP1 blocks transcriptional activation of NRF2’s target genes by preventing NRF2 from accessing the nucleus. Furthermore, while bound, KEAP1 targets NRF2 for ubiquitination and subsequent proteasome degradation. At baseline, NRF2 abundance in the cytoplasm is low. However, under conditions of oxidative stress, cysteine residues on KEAP1 are modified, inhibiting ligase activity. In this state, NRF2 begins to accumulate in the cytoplasm. Eventually, a portion of this accumulating NRF2 protein begins to localize into the nucleus [25]. Once in the nucleus, NRF2 binds to antioxidant response elements (ARE), causing transcription of multiple genes involved in combating oxidative stress [26]. Recent studies have suggested that NRF2 may have both neuroprotective as well as anti-aging effects on cells [27–32]. Interestingly, recent work has shown a critical role for NRF2 in regulating iron metabolism, lipid peroxidation, and ferroptosis [33, 34]. In particular, NRF2 has been implicated in the repression of ferroptosis through its indirect regulation of GPX4 [35–39], as well as its direct transcriptional regulation of critical antioxidant proteins such as SLC7A11 (sometimes referred to as the “NRF2/SLC7A11” axis) [40–42]. Therefore, NRF2 activators are promising candidates for the treatment of conditions where ferroptosis is a known pathology, particularly for cases where SLC7A11 or “system X_c_^-^” are being inhibited (e.g. class I ferroptosis inducers).

Since its discovery, the mitochondrial role in ferroptosis has been uncertain. Initial experiments suggested the mitochondria were not required for the progression of ferroptosis, despite significant morphological changes observed in the mitochondrial network during end-stage ferroptosis [12, 43]. However, in 2019, Gao et al. demonstrated that mitochondria-depleted cells were less prone to ferroptosis triggered by erastin or cysteine depletion, suggesting an integral role of the mitochondrial network in the ferroptosis pathway [44]. Another recent report has shown mitochondrial ROS (mtROS) to be an important trigger for ferroptosis, and that mitophagy (mitochondrial-specific autophagy) is a key mechanism for keeping mtROS levels low in normal cells [45]. Following from this, experiments using a novel inner mitochondrial membrane (IMM)-targeted fluorescent lipid peroxidase probe, termed MitoCLox, revealed that lipid peroxidation of the IMM occurs in vitro during erastin-induced ferroptosis, further supporting the involvement of lipid peroxidation of the mitochondrial membrane in ferroptosis [46]. More recent work has sought to further explore the role of mitophagy and “ferritinophagy” (the selective degradation or ferritin to release free iron) in regulating ferroptosis across various forms of disease [47–51].

The iron-related gene *FDXR* encodes ferredoxin reductase (FDXR), a flavoprotein localized to the IMM. FDXR plays an important role in both steroid hormone synthesis as well as ISC biogenesis in the mitochondria. In these pathways, FDXR is responsible for transferring electrons from NADPH to ferredoxin proteins, FDX1 and FDX2 [52, 53]. Although the tissue expression profiles of the two ferredoxin proteins are distinct, both appear to be important for ISC formation [54]. This participation in ISC biogenesis places FDXR and FDX1/2 in the same metabolic pathway as frataxin (FXN), the protein associated with the iron overload disorder FRDA. ISCs are cofactors required for the function of multiple proteins and are essential to many cellular functions [55, 56], with several of the proteins involved in the synthesis of ISCs conserved from prokaryotes to eukaryotes [57–63].

In 2017, our group was among the first to report that biallelic, loss-of-function variants in the *FDXR* gene resulted in mitochondriopathy in humans [64]. Clinically, FDXR-related mitochondriopathy is characterized by optic atrophy, neurological dysfunction, movement disorder, hearing loss, developmental regression, and/or global developmental delays [64, 65]. Interestingly, approximately 25% of FDXR-related mitochondriopathy is caused by a single hotspot variant in either the homozygous or compound heterozygous state, with an estimated allele frequency 1/185 in the Mexican population [66]. This hotspot variant is located at amino acid 386, causing an amino acid substitution of arginine to tryptophan (NM_024417.5: c.1156C>T, p.Arg386Trp,). The arginine at this amino acid position is very highly conserved across mammals, reptiles, and fish, as we have previously shown [64], suggesting that it is likely to be very important to FDXR protein function. Due to this noticeably high allele frequency at such a well-conserved amino acid position, we sought to generate a mouse model with a corresponding genotype. Interestingly, a pre-existing mouse model from Jackson Lab possesses a spontaneously occurring variant in the *Fdxr* gene at the analogous location to the human hotspot variant, termed “R389Q” (*B6;129S-Fdxr*^*m1J*^ *Otop2*^*m1J*^*/GrsrJ*, Stock #026096). We have previously characterized these mice across several publications [10, 64, 67]. Despite the amino acid substitution differing from the human hotspot (glutamine vs. tryptophan), these spontaneously occurring mice demonstrated optic atrophy, retinal ganglion cell (RGC) loss, and movement disorders [68]. Additionally, iron studies in *Fdxr*^R389Q/R389Q^ mice showed mitochondrial iron overload and defects in mitochondrial membrane potential (MMP) [10].

However, while these homozygous Fdxr^R389Q/R389Q^ mice do show a neurological phenotype and a stiffening of the hindquarters similar to patients with FDXR-related mitochondriopathy, there are several factors that limit the utility of this animal model. For one thing, the severity of the phenotype observed in the Fdxr^R389Q/R389Q^ mice is noticeably mild compared to the crippling ataxia, severe weight loss, and poor overall health observed in FDXR patients (and particularly those patients with the homozygous p.R386W variants). This makes sense when one considers that arginine and glutamine are much more chemically similar to each other than arginine and tryptophan. Thus, one might naturally predict that the arginine to tryptophan substitution observed in human patients would result in a much more severe phenotype than the arginine to glutamine substitution observed in Jackson Lab’s existing *Fdxr*^R389Q/R389Q^ mouse line. In addition, the existing “R389Q” mouse line contains an additional mutation in otopetrin 2 (Otop2), which may be contributing to the phenotype observed in these mice. Moreover, to our knowledge, the p.R386Q variant has yet to be described in individuals with FDXR-related mitochondriopathy, suggesting that the *Fdxr*^R389Q/R389Q^ mouse line is not the best possible model for the human disease.

Therefore, to better model the human disease, we have generated knock-in mice carrying the corresponding amino acid substitution (“R389W”) to the human hotspot variant. In this paper, we will first describe this new mouse model of FDXR-related mitochondriopathy (*Fdxr*^*R389W/R389W*^) and offer insights on the resulting phenotype. Using this model, we will show that ferroptosis is an underlying molecular pathology in patient-derived cells. Furthermore, we will demonstrate that the NRF2/SLC7A11 antioxidant response pathway, which is critical in inhibiting ferroptosis in several biological contexts [40, 69, 70], is downregulated in FDXR-related mitochondrial disease. Finally, we will show that the new NRF2-activating drug *omaveloxolone* (also known as RTA-408, or Skyclarys®) can improve dysfunction related to ferroptosis by upregulating this important NFR2/SLC7A11 axis.

## Results

### Creation and phenotypic characterization of the *Fdxr*^*R389W/R389W*^ mouse

**Fig. 1:**
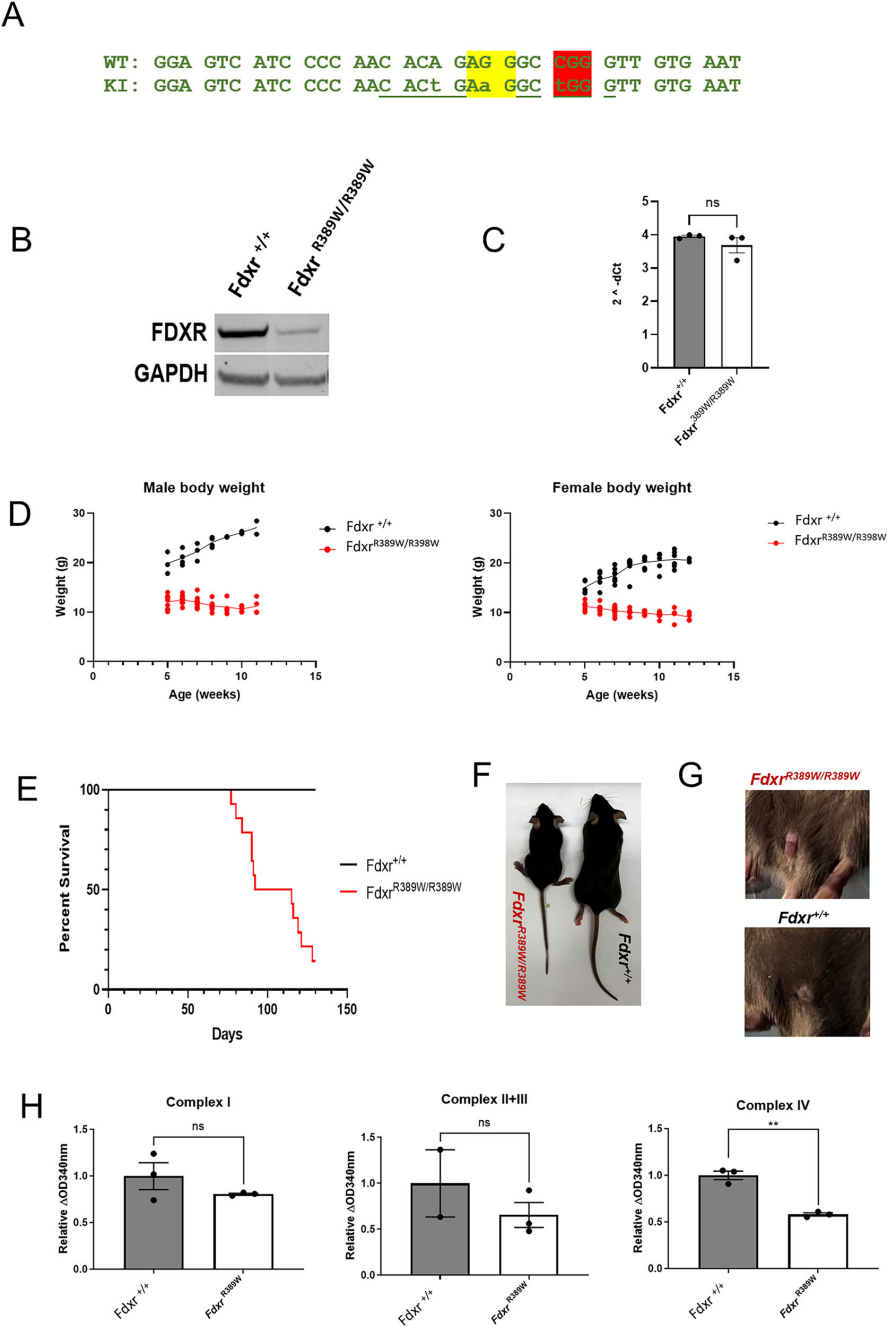
Creation and phenotypic characterization of the *Fdxr*^*R389W/R389W*^ mouse. **A** Nucleotide mutations generated in the *Fdxr*^*R389W/R389W*^ knock-in (KI) mouse model, as compared to the wildtype (WT) sequence. The sequence highlighted in yellow represents the protospacer adjacent motif (PAM) required for Cas9 targeting, and sequence highlighted in red represents codon 389 (where the arginine to tryptophan coding change is made). The underlined sequences represent TspRI (CACTGA) and BseYI (GCTGGG) digest sites for distinguishing the WT and mutant alleles. **B** Western blot showing protein levels of FDXR in the adrenal gland of 2-month old *Fdxr*^*R389W/R389W*^ and *Fdxr*^*+/+*^ littermates. **C** Expression of *Fdxr* transcript in the adrenal gland of 2-month old *Fdxr*^*R389W/R389W*^ and *Fdxr*^*+/+*^ littermates, as determined by qRT-PCR. Statistical testing was performed using a nonparametric, unpaired, two-tailed Mann-Whitney U test. Results are presented as means ± SEM.; ns: not significant. **D** Weight of male and female of *Fdxr*^*R389W/R389W*^ and *Fdxr*^*+/+*^ littermates in weeks. **E** Survival curve of *Fdxr*^*R389W/R389W*^ and *Fdxr*^*+/+*^ littermates, showing a significantly shortened lifespan for *Fdxr*^*R389W/R389W*^ mutant mice (p < 0.0001 based on the Mantel-Cox test; n = 14 for *Fdxr*^*+/+*^ WT mice, and n = 9 for *Fdxr*^*R389W/R389W*^ mutant mice). **F** Photo showing body habitus and stiffened/splayed hindlimbs of a *Fdxr*^*R389W/R389W*^ mouse relative to WT littermate. **G** Photo of paraphimosis phenotype in an 11-week old *Fdxr*^*R389W/R389W*^ male mouse. An age-matched, WT male sibling is also shown for comparison. **H** Relative activity of complex activities in the mitochondria of *Fdxr*^*R389W/R389W*^ and *Fdxr*^*+/+*^ littermates. Statistical testing was performed using two-tailed, unpaired Student’s t-tests. Results are presented as means ± SEM.; **p < 0.01; NS: not significant.

To better understand FDXR*-*related mitochondriopathy caused by the human FDXR hotspot variant, a knock-in mouse model at the analogous location (p.R389W) was generated using CRISPR/Cas9 (Fig. 1A). The genotype of the resulting *Fdxr*^*R389W/R389W*^ mice was confirmed via PCR and Sanger sequencing. Our previous study showed the protein level was reduced in patients homozygous for this hotspot variant [64]. As shown in Fig. 1B, FDXR protein concentration was drastically reduce in the adrenal gland of *Fdxr*^*R389W/R389W*^ mice, supporting our previous results in human tissues (Fig. 1B). Interestingly, mRNA expression was unchanged in mutants, suggesting that the R389W mutation affects protein stability rather than transcription (Fig. 1C). Phenotypically, *Fdxr*^*R389W/R389W*^ mice had decreased weight and significantly reduced survival relative to wildtype littermates, with a median life expectancy of 90 days (Fig. 1D, E). Moreover, *Fdxr*^*R389W/R389W*^ mice had a sickly body habitus, which was easily differentiated from wild-type littermates (Fig. 1F). In addition, male *Fdxr*^*R389W/R389W*^ mice frequently exhibited paraphimosis with age (Fig. 1G), a condition previously reported in other mouse models of neurological disease [71, 72].

In consideration of FDXR function in the mitochondria, we also explored ETC complex activities using heart-derived mitochondrial extracts generated from *Fdxr*^*R389W/R389W*^ mutants. Not surprisingly, all complex activities were depressed relative to wild-type littermates, but most significantly for complex IV activity (Fig. 1H). Together, this data supports that *Fdxr*^*R389W/R389W*^ mice recapitulate the severe phenotype in patients carrying the hotspot variant.

### Neurologic and ophthalmological phenotypes in *Fdxr*^*R389W/R389W*^ mice

**Fig. 2:**
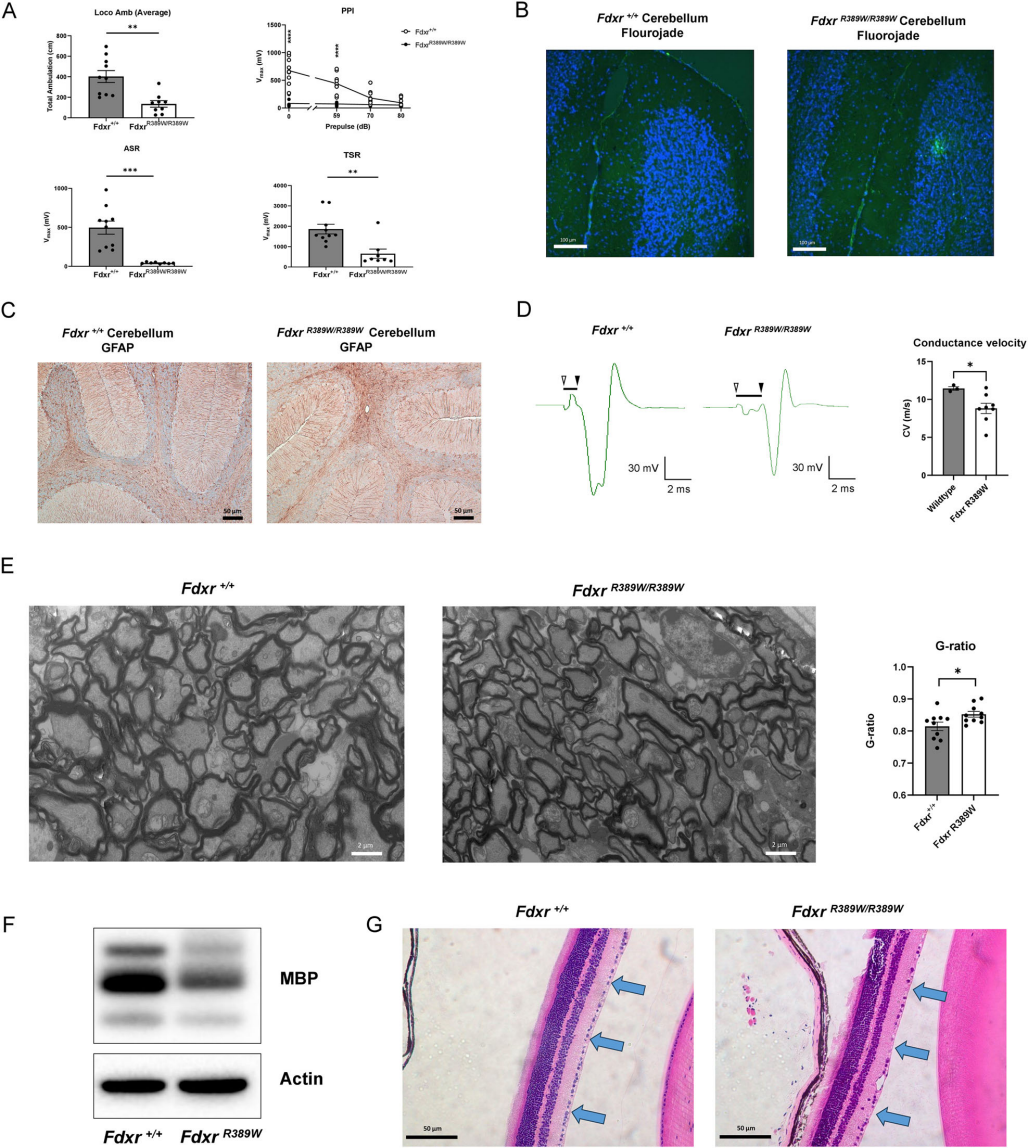
Motor impairment, neurological defects and ophthalmological disease in the *Fdxr*^*R389W/R389W*^ mutant mice. **A** Behavioral tests to assess movement were performed in *Fdxr*^*R389W/R389W*^ mice and Fdxr^+/+^ littermates including locomotor ambulation (Loco Amb), pre-pulse inhibition (PPI) presented as startle amplitude (Vmax) measured in units of voltage change (mV), tactile startle response (TSR) and acoustic startle response (ASR). For all behavioral assays, n = 8–10 animals per genotype. For all results, statistical testing was performed using nonparametric, unpaired, two-tailed Mann-Whitney U tests. For PPI results, the Holm-Šídák method was also used to adjust for multiple testing correction. In addition, histological staining of cerebellum tissue sections from 2 month old mutant and control mice with Fluoro-jade-C (**B**) and anti-GFAP (**C**) showed evidence of increased neurodegeneration and gliosis, respectively, in mutant tissues. Scale bars: 100 μm for panel B and 50 μm for (**C**). **D** Furthermore, electromyograms (EMGs) recorded from the gastrocnemius muscle upon electrical stimulation of the sciatic nerve showed reduced conduction velocity in mutant mice consistent with compromised motor neuron function (scale on bottom right indicates voltage and time). For each EMG trace, the onset of the stimulus is indicated by an open arrowhead, the onset of the response is indicated with a closed arrowhead, and the latency between the stimulus and the response is indicated with a black bar. For conductance velocity results, statistical testing was performed using the two-tailed, unpaired Student’s t-test. **E** To assess eye pathology, transmission electron micrographs (TEMs) were performed on optic nerve fibers from 6-month-old *Fdxr*^*R389W/R389W*^ mice and *Fdxr*^*+/+*^ littermates. G-ratio, or the ratio between the inner axon diameter and the total axon fiber diameter, was also assessed for optic nerve fibers from these images. The results indicate a significant thinning of the myelin layer in mutant optic nerves relative to wildtype controls. For G-ratio results, statistical testing was performed using the two-tailed, unpaired Student’s t-test. Scale bar: 2 μm. **F** MBP (myelin basic protein) was also examined in the optic nerves of wildtype and *Fdxr*^*R389W/R389W*^ mutant mice using western blotting, demonstrating loss of MBP in the *Fdxr*^*R389W/R389W*^ mutant mice. (**G**) Finally, H&E-staining was performed on retinal tissues sections. The results showed a distinctly reduced number of RGCs in the ganglion cell layer (blue arrows) in sections of eyes from 2-month-old *Fdxr*^*R389W/R389W*^ and *Fdxr*^*+/+*^ littermates. Scale bar: 50 μm. For all panels in this figure, statistical results are presented as means ± SEM.; *p < 0.05, **p < 0.01, ***p < 0.001, ****p < 0.0001.

As optic atrophy, retinal disease, and neurological dysfunction are common findings in individuals impacted by FDXR-related mitochondriopathy, we sought to evaluate these phenotypes in *Fdxr*^*R389W/R389W*^ mice utilizing locomotor behavioral assessments and histological analysis, respectively. Mutant mice had significantly decreased locomotor ambulation relative to wild-type littermates (P < 0.001), confirming a substantial movement disorder in mutants (Fig. 2A). Further analysis revealed signs of depression of the autonomic nervous system and sensorimotor gating in *Fdxr*^*R389W/R389W*^ mutants (Fig. 2A). Histological staining of the brain revealed further evidence of neurological damage in *Fdxr*^*R389W/R389W*^ mutant mice as indicated by increased Fluoro-Jade C staining (Fig. 2B), a widely used fluorescent dye for staining degenerating neurons. *Fdxr*^*R389W/R389W*^ mutant brain tissue also showed increased staining for Glial Fibrillary Acidic Protein (GFAP), indicating the presence of increased reactive gliosis and tissue damage in the *Fdxr*^*R389W/R389W*^ mutant brain tissue (Fig. 2C). EMG recording from the gastrocnemius muscle of mutants also indicated a decreased conduction velocity (Fig. 2D), consistent with compromised motor neuron function in mutants.

Next, given the well-characterized optic atrophy phenotype also observed in patients with FDXR-related mitochondriopathy, we assessed eye tissue in *Fdxr*^*R389W/R389W*^ mutant mice using histological analysis. Transmission electron microscopy (TEM) imaging of the optic nerve showed observable defects in myelination and axon morphology, including an apparently smaller size and diameter in the mutant axons relative to the wildtype axons. (Fig. 2E). *Fdxr*^*R389W/R389W*^ mice also had significantly higher g-ratios relative to wildtype littermates (P < 0.05) suggesting abnormal myelination in the optic nerve (Fig. 2E). However, g-ratio calculations alone may be misleading, particularly if the axon diameters of the mutant and wildtype optic nerve samples are not the same. Therefore, we also carried out a western blot of myelin basic protein (MBP) levels in wildtype and mutant optic nerve tissue to provide an additional validation for this result. Consistent with an overall thinning and loss of myelin sheath mass, MBP level was reduced in the optic nerve of mutant mice relative to control mice (Fig. 2F). Lastly, H&E staining of the retina revealed a decreased number of RGCs in the ganglion cell layer (Fig. 2G), consistent with retinal degeneration, a common finding in FDXR patients [64–66, 68, 73–77]. Together, these data demonstrate that *Fdxr*^*R389W/R389W*^ mice better mimic the neurological and ophthalmological phenotype observed in humans with FDXR-related mitochondriopathy relative to the *Fdxr*^*R389Q/R389Q*^ model.

### Mitochondrial iron accumulation in FDXR deficient cells is associated with membrane lipid peroxidation and increased susceptibility to ferroptosis

**Fig. 3:**
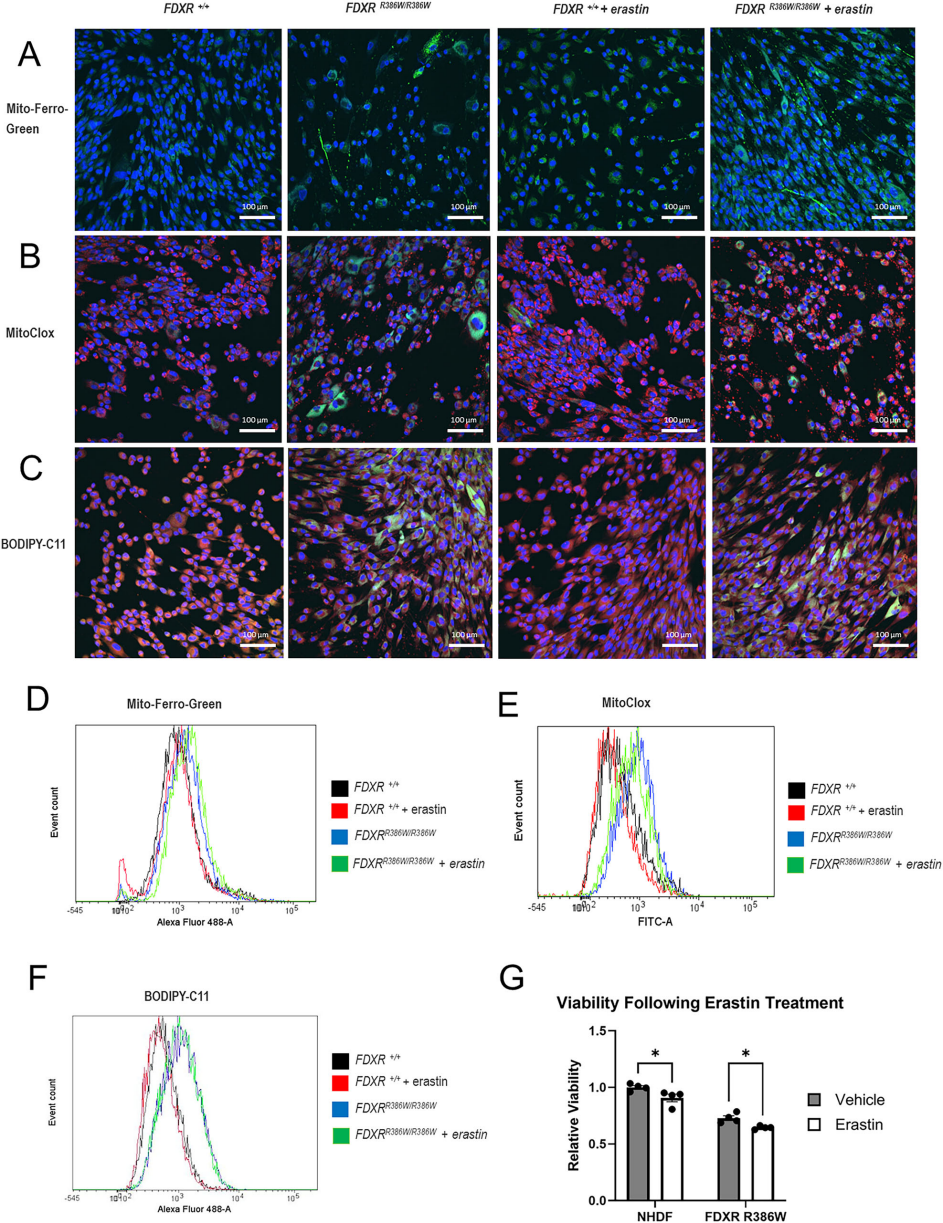
Participant-derived fibroblasts with a *FDXR*^R386W/R386W^ genotype are more susceptible to ferroptosis with or without erastin-induction. Fibroblasts were cultured with or without erastin (5 μM) for 15 h. After incubation, cells were stained with varying fluorophores and analyzed by fluorescent microscopy (**A–C**) or Flow cytometry as measured in mean fluorescent intensity (MFI) (**D–F**). Fluorophores used included Mito-Ferro-Green (**A**, **D**), MitoCLox (**B**, **E**), or BODIPY-C11 (**C**, **F**). Scale bars: 100 μm for panels A-C. **G** Cell viability was also examined in control and *FDXR*^R386W/R386W^ cells, with and without erastin treatment, using an XTT viability assay kit. Statistical testing was performed using nonparametric, unpaired, two-tailed Mann-Whitney U tests. Results are presented as means ± SEM.; *p < 0.05.

Recently, our group demonstrated that FDXR deficient cells had mitochondrial iron overload [10]. As iron overload has been associated with ferroptosis in other disorders of ISC deficiency, we explored this pathogenesis in *FDXR*^*R386W/R386W*^ patient-derived fibroblasts (also referred to as *FDXR*^*R386W/R386W*^ cells) [19]. To do so, we performed Prussian blue staining and found iron accumulation in *FDXR*^*R386W/R386W*^ cells (Supplementary Fig. S1). Further, iron accumulation in the mitochondria of *FDXR*^*R386W/R386W*^ cells was observed via immunocytochemistry (ICC) using Mito-Ferro-Green, a fluorophore which targets mitochondrial Fe^2+^ (Fig. 3A). Analysis was performed with and without erastin induction to determine the impact of ferroptosis on iron overload. Higher levels of Mito-Ferro-Green fluorescence were observed in *FDXR*^*R386W/R386W*^ cells relative to NHDF control, suggesting increasing levels of mitochondrial Fe^2+^ in the *FDXR*^*R386W/R386W*^ cells. Fe^2+^ was also more frequent in erastin-induced cells, suggesting that ferroptosis exacerbates mitochondrial iron overload. Consistent with this, the expression of multiple iron metabolism-related genes was also found to be altered in *FDXR*^*R386W/R386W*^ cells (Supplementary Fig. S2). Flow cytometry also confirmed a higher mean fluorescent intensity (MFI) in *FDXR*^*R386W/R386W*^ cells (Fig. 3D), supporting our previous work showing iron overload in mitochondrial extracts of *Fdxr*^*R389Q/R389Q*^ mice [10]. Corresponding to ICC staining, MFI confirmed mitochondrial iron accumulation was highest in erastin-induced *FDXR*^*R386W/R386W*^ cells. Due to the increase in the mitochondrial labile iron pool in *FDXR*^*R386W/R386W*^ cells, we then questioned whether iron overload leads to ROS production and lipid peroxidation of IMM. Using the fluorophore MitoCLox, we assessed lipid peroxidation in IMM via live-cell ICC staining and flow cytometry. MitoCLox is a fluorescent fluorophore sensitive to lipid peroxidiation that is relatively specific for the IMM. MitoCLox was originally designed with a long linker containing two peptide bonds that mimicked the artificial tetrapeptide SS-20, which allows MitoCLox to target the inner mitochondrial membrane via an affinity for the IMM lipid cardiolipin (or “CL”) [78, 79]. ICC staining with MitoCLox showed a distinct increase in IMM lipid peroxidation in *FDXR*^*R386W/R386W*^ cells with and without erastin induction (Fig. 3B). Flow cytometry also confirmed that MFI was higher in *FDXR*^*R386W/R386W*^ cells, even without erastin induction (Fig. 3E). Interestingly, IMM lipid peroxidation was observed more strongly in non-induced cells relative to erastin-induced cells, which is likely a reflection of the shrunken and altered mitochondrial morphology that has been previously observed in ferroptotic cells [12, 80].

Having established the presence of IMM lipid peroxidation in *FDXR*^*R386W/R386W*^ cells, we further tested the susceptibility of *FDXR*^*R386W/R386W*^ cells to erastin-induced ferroptosis by measuring lipid peroxidation events in all lipid membranes, including the plasma membrane, a hallmark feature of ferroptotic cells [18]. Interestingly, prior to induction with erastin, *FDXR*^*R386W/R386W*^ cells had a higher MFI peak than controls, again showing that lipid peroxidation of lipid membranes is present at baseline for this genotype. After induction with erastin, *FDXR*^*R386W/R386W*^ cells continued to demonstrate membrane lipid peroxidation and increased cell death relative to normal human dermal fibroblast control cells (also referred to as “NHDF” cells) (Fig. 3C, F). Increased lipid peroxidation was also observed in other cell lines derived from FDXR-related mitochondriopathy patients, confirming that ferroptosis predisposition is not specific to the hotspot variant (Supplementary Fig. S3). Ultimately, all of these ferroptosis-related features observed in *FDXR*^*R386W/R386W*^ cells would be expected to affect cell viability. We therefore examined cell viability using XTT colorimetric assays. We found that the viability of *FDXR*^*R386W/R386W*^ cells were reduced at baseline relative to NHDF cells (Supplementary Fig. S4), and that erastin decreased the viability in both NHDF cells and *FDXR*^*R386W/R386W*^ cells (Fig. 3G).

### The NRF2 pathway and the ferroptosis inhibitor SLC7A11 are depleted in FDXR^R386W/R386W^, and can be restored by pharmaceutical activation of NRF2

**Fig. 4:**
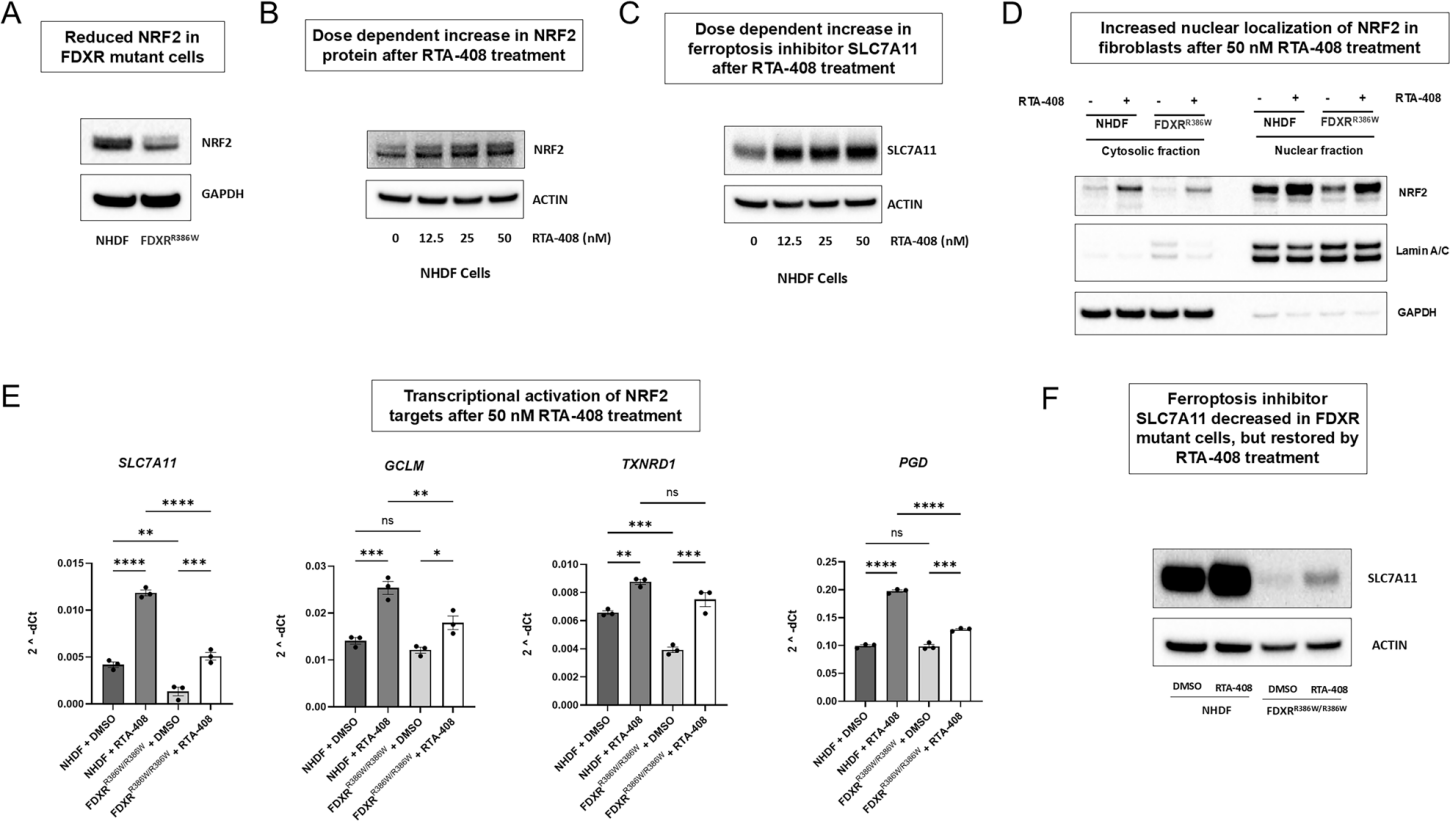
*FDXR*^R386W/R386W^ cells display significant reductions in NRF2 and SLC7A11, which can be restored following treatment with the NRF2 activator omaveloxolone (RTA-408). **A** NRF2 protein levels are noticeably reduced in *FDXR*^R386W/R386W^ cells. NHDF cells show a dose-dependent increase in NRF2 protein (**B**) as well as the NRF2 transcriptional target SLC7A11 (**C**) in response to varying concentrations of RTA-408. **D** Western blot analysis of nuclear and cytoplasmic extracts from *FDXR*^R386W/R386W^ cells and NHDF cells treated with omaveloxolone. The results demonstrate increased expression and nuclear localization of NRF2 following omaveloxolone treatment in both *FDXR*^R386W/R386W^ cells as well as NHDF cells. Anti-GAPDH staining is used as a loading control for cytoplasmic fractions (left), while anti-lamin antibody staining is used as a loading control for nuclear fractions. **E** As a result of increased NRF2 protein levels and translocation of NRF2 to the nucleus, NRF2 transcriptional targets also show increased expression following RTA-408 treatment in both *FDXR*^R386W/R386W^ cells as well as NHDF cells. Statistical testing was performed using a One-way ANOVA omnibus test, followed by post hoc testing using Tukey’s multiple comparisons test to determine individual p-values. Results are presented as means ± SEM.; *p < 0.05, **p < 0.01, ***p < 0.001, ****p < 0.0001, NS: not significant. **F** Finally, western blot analysis of *FDXR*^R386W/R386W^ cells confirms that they show a drastic reduction SLC7A11 protein level relative to NHDF cells, and that the level SLC7A11 protein is partially restored following treatment with RTA-408.

As shown in Fig. 3, *FDXR* mutations are associated with increased lipid peroxidation and ferroptosis. Since ferroptosis appears to be a probable pathological mechanism of FDXR-related mitochondriopathy, we next aimed to mitigate the effects using chemical inhibitors of ferroptosis. As one initial option, we targeted the “class IV” mechanism of ferroptosis (mediated by the labile iron pool) using the iron chelator deferoxamine (DFO), but found that DFO significantly reduced cell viability in NHDF cells (Supplementary Fig. S5). As another option, we also tested a chemical activator of NRF2. Since NRF2 activators have been shown to be an effective and safe treatment for humans with FRDA (another iron overload disorder), we investigated the role of NRF2 in *FDXR*^*R386W/R386W*^ cells. We found that NRF2 level was considerably reduced in *FDXR*^*R386W/R386W*^ cells (Fig. 4A), similar to what is observed in FRDA. This suggests that NRF2 activation could be beneficial for FDXR-related mitochondriopathy, as it has already proven beneficial for the mechanistically similar disease FRDA.

Omaveloxolone, a recently FDA approved NRF2 activator, has been shown to improve neurological function in individuals with FRDA via modulation of NRF2 [81], likely by activating downstream antioxidant genes [82–85]. Moreover, NRF2 induction has been shown to decrease susceptibility to ferroptosis in FXN-deficient cells, suggesting omaveloxolone may also be effective at mitigating the effects of ferroptosis in FDXR mutant cells [86]. In order to establish the optimal dose for NRF2 induction, NHDF cells were first tested with varying doses of omaveloxolone. As expected, the results showed a dose-dependent increase in the NRF2 protein (Fig. 4B) as well as the NRF2 transcriptional target and ferroptosis inhibitor SLC7A11 (Fig. 4C).

Additional analysis from *FDXR*^*R386W/R386W*^ cells demonstrated increased expression and nuclear localization of NRF2 following omaveloxolone treatment (Fig. 4D), as well as increased transcription of NRF2 target genes (Fig. 4E). Western blot and qRT-PCR also confirmed that *FDXR*^R386W/R386W^ cells show a drastic reduction in SLC7A11 protein (Fig. 4F) protein, as well as mRNA levels for *Slc7a11* and other NRF2 target genes (Fig. 4E). The results also show that SLC7A11 and multiple other NRF2 target genes are partially restored to normal levels following omaveloxolone treatment (Fig. 4E, F). Interestingly, two of the iron-metabolism genes shown in Supplementary Fig. S2 that are also NRF2 targets, *HMOX1* and *SLC40A1*, were actually increased in *FDXR*^R386W/R386W^ cells. However, previous research has shown that *HMOX1* can be activated by both NRF2-dependent and NRF2-independent mechanisms [87, 88], and multiple publications have also indicated that NRF2 may actually repress the transcription of *SLC40A1 (ferroportin*) in certain biological contexts [34, 89]. Thus, it appears likely that HMOX1 and SLC40A1 are being activated by NRF2 independent mechanisms in *FDXR*^*R386W/R386W*^ cells, likely induced by the presence of increased iron in the patient cells. We also attempted to examine whether the OMA1-DELE1 mitochondrial stress pathway was activated in *FDXR*^*R386W/R386W*^ cells, and observed a potential cleaved DELE1 band (about 29 kD) that was considerably increased in *FDXR*^*R386W/R386W*^ cells (Supplementary Fig. S6). However, this last result with DELE1 may require further validation in the future as this and other anti-DELE1 antibodies have often shown inconsistent results based on the existing literature in the field [90, 91].

**Fig. 5:**
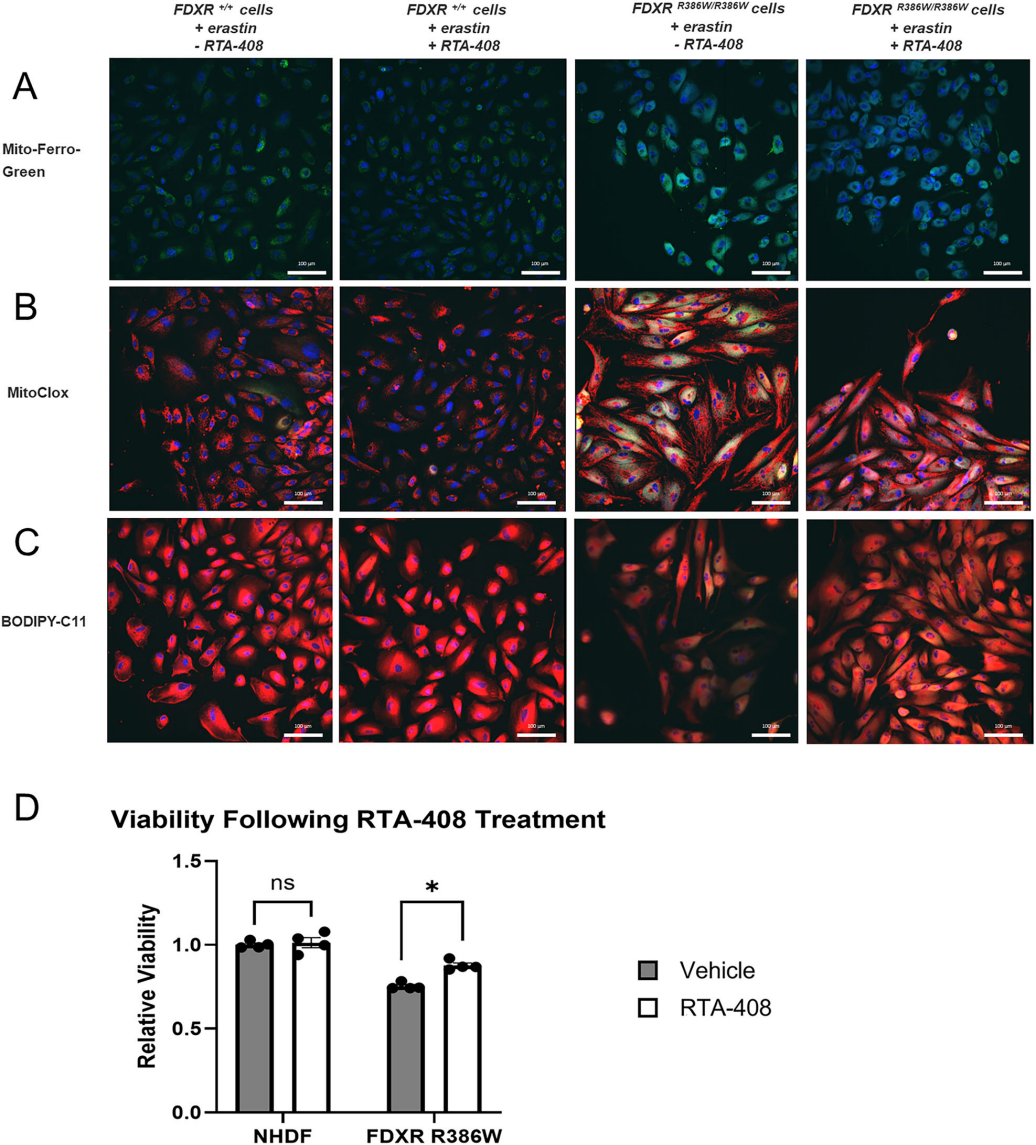
Participant-derived fibroblasts with a *FDXR*^R386W/R386W^ genotype are less susceptible ferroptosis, membrane lipid peroxidation, and iron overload after RTA-408 treatment. Fibroblasts were cultured for 48 h with RTA-408 or vehicle-only control (DMSO), and then treated with erastin (5 μM) for 15 h. After incubation, cells were stained with either Mito-Ferro-Green (**A**), MitoCLox (**B**), or BODIPY-C11 (**C**), and imaged by fluorescent microscopy. Scale bars: 100 μm for (**A**–**C**). **D** Cell viability was also examined in control and FDXR patient fibroblasts, with and without RTA-408 treatment, using an XTT viability assay kit. Statistical testing was performed using nonparametric, unpaired, two-tailed Mann-Whitney U tests. Results are presented as means ± SEM; ns not significant, *p < 0.05.

Finally, fluorescent microscopy analysis showed that *FDXR*^R386W/R386W^ cells are less susceptible to erastin-induced ferroptosis, membrane lipid peroxidation, and iron overload following omaveloxolone treatment (Fig. 5A–C). Most crucially, omaveloxolone treatment also increased viability in *FDXR*^R386W/R386W^ cells (Fig. 5D). Overall, this loss of NRF2/SLC7A11 expression and its restoration by omaveloxolone suggests that ferroptosis via the “class I” mechanism (i.e. inhibition of the system X_c_^-^/GSH pathway) is likely to be occurring in FDXR-related mitochondriopathy. Taken together, we propose that omaveloxolone may be beneficial for alleviating symptoms in FDXR-related mitochondriopathy secondary to ferroptosis via stimulation of the NRF2 pathway, by activating the NRF2/SLC7A11 axis and helping to partially restore the system X_c_^-^/GSH pathway.

### Activation of NRF2-dependent stress response pathways with omaveloxolon*e* mitigates the pathogenic effects of *Fdxr*^*R389W/R389W*^ mutation in mice

**Fig. 6:**
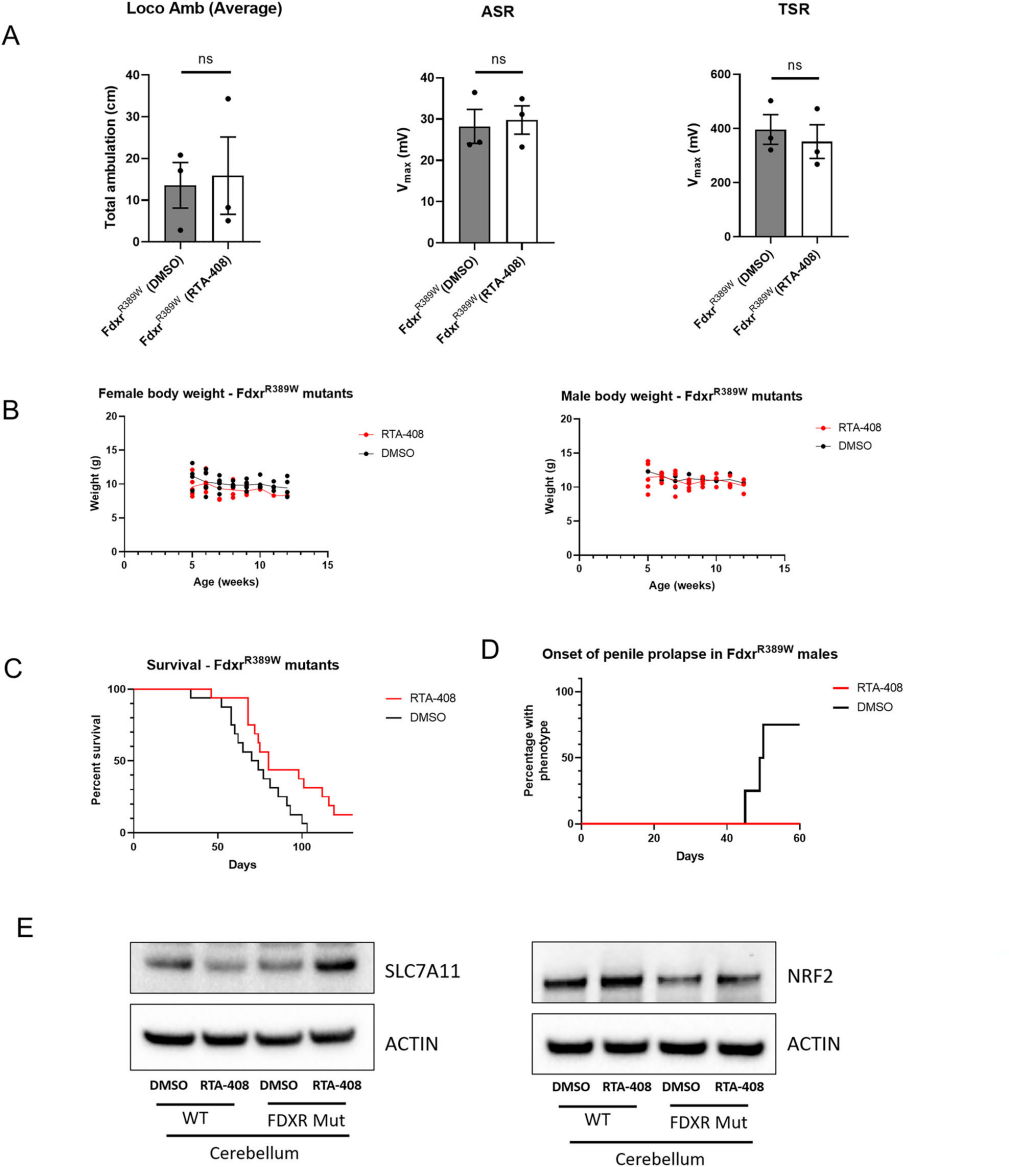
Activation of NRF2 response pathways with RTA-408 partially mitigates the pathogenic effects of *Fdxr* mutation in *Fdxr*^R389W/R389W^ mice. *Fdxr*^*R389W/R389W*^ mice were treated with daily injections of 17.5 mg/kg RTA-408”, or vehicle only control (“DMSO”). **A** In behavioral testing, RTA-treated and DMSO-treated *Fdxr*^*R389W/R389W*^ mutants showed no significant differences in locomotor ambulation (Loco Amb), tactile startle response (TSR) and acoustic startle response (ASR) (n = 3 mice per treatment). Statistical testing was performed using two-tailed, unpaired Student’s t-tests. Results are presented as means ± SEM.; NS not significant. **B** Similarly, there was no improvement in weight for RTA-408 treated male or female mice as compared to DMSO-treated mice (n = 3–8 mice per treatment for each sex). **C** In contrast, *Fdxr*^*R389W/R389W*^ mice treated with RTA-408 injections did show an overall improvement in survival through 130 days of treatment relative to DMSO controls (p = 0.0248 based on the Mantel-Cox test, n = 16 mice per treatment). **D** In addition, the onset of the paraphimosis (penile prolapse) in *Fdxr*^R389W/R389W^ male mice was completely stopped through at least 60 days of age following RTA-408 treatment, while around 75% of DMSO treated mice display this phenotype by the same age (p = 0.0221 based on the Mantel-Cox test, n = 4–5 male mice per treatment). **E** These phenotypic improvements are further supported by western blot analysis of NRF2 and SLC7A11 in brain (cerebellum) of male *Fdxr*^*R389W/R389W*^ and male *Fdxr*^*+/+*^ mice treated with either RTA-408 or DMSO. Consistent with the results from the *FDXR*^R386W/R386W^ cells, western blot analysis shows that both NRF2 and SLC7A11 are reduced at baseline in DMSO-treated mutants relative to DMSO-treated wildtype mice, and that RTA-408 treatment dramatically increases the levels of both proteins in *Fdxr*^*R389W/R389W*^ mutants.

We next explored the in vivo therapeutic effects of omaveloxolone on FDXR pathology using our novel *Fdxr*^*R389W/R389W*^ mouse model. Following daily injections of 17.5 mg/kg omaveloxolone, *Fdxr*^*R389W/R389W*^ mutants were examined for critical phenotypes such as behavior, weight, and survival. While omaveloxolone-treated *Fdxr*^*R389W/R389W*^ mutants showed no significant improvements in motor performance (Fig. 6A) or weight gain (Fig. 6B) relative to DMSO-treated controls, improvements were observed for other important health measures. Importantly*, Fdxr* mutants showed a significant improvement in survival through 130 days of treatment (Fig. 6C). In addition, the onset of paraphimosis commonly observed in untreated *Fdxr*^R389W/R389W^ male mice was completely stopped through at least 60 days of age following omaveloxolone treatment (Fig. 6D). These phenotypic improvements in *Fdxr*^*R389W/R389W*^ mutants are further supported by western blot analysis of NRF2 and SLC7A11 (Fig. 6E). Consistent with the results from the *FDXR*^*R386W/R386W*^ cells showing reduced NRF2 protein and SLC7A11 protein at baseline (Fig. 4A, D, F), western blot analysis of cerebellum tissue from our *Fdxr* mouse model also shows that both NRF2 and SLC7A11 are reduced at baseline in DMSO-treated *Fdxr*^*R389W/R389W*^ mutants relative to DMSO-treated wildtype mice (Fig. 6E). This demonstrates that reduced NRF2 and SLC7A11 are a consistent feature of FDXR dysfunction across species. Furthermore, western blot shows that omaveloxolone treatment increases the levels of both NRF2 and SLC7A11 in *Fdxr*^*R389W/R389W*^ mutant tissue (Fig. 6E), as observed in *FDXR*^*R386W/R386W*^ cells (Fig. 4D, F).

Taken together, the mouse and patient fibroblast data clearly demonstrate that loss-of-function mutations in the *FDXR* gene result in ferroptosis associated with loss of NRF2 and its important downstream targets such as SLC7A11. Moreover, the results show that pharmacological upregulation of NRF2 in FDXR-deficient cells inhibits ferroptosis and ameliorates disease symptoms in a mouse model of FDXR-mitochondriopathy.

## Discussion

**Fig. 7:**
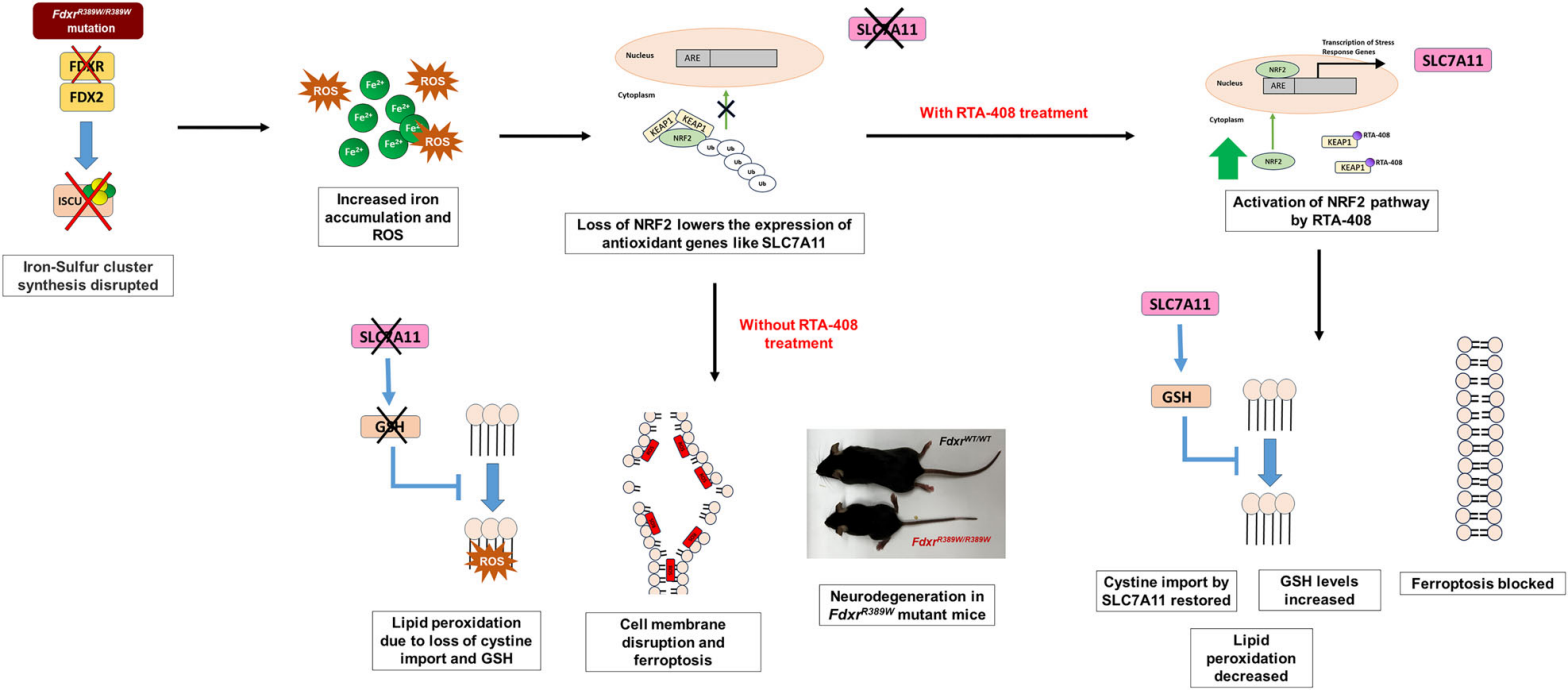
Proposed mechanism of FDXR loss of function triggering ferroptosis. In the proposed model, loss of FDXR leads to a decrease in ISC formation. ISC deficiency, in turn, is expected to not only to impact ISC-dependent complexes in the electron transport chain (causing ATP depletion), but as ISC biosynthesis is suppressed, iron accumulates in the mitochondria, further increasing ROS production via Fenton reaction intermediates. This iron-based ROS accumulation leads to IMM lipid peroxidation as well as slow depletion of GSH and antioxidant defenses. At the same time, the loss of NRF2 protein lowers the expression of antioxidant genes and key ferroptosis inhibitors, such as the cystine/glutamate antiporter SLC7A11. Together, this increased mitochondrial iron accumulation and decreased function of NRF2/SLC7A11 leads to a deficient redox response and excessive lipid peroxidation of the plasma membrane, resulting in ferroptosis. Finally, pharmacological activation of the NRF2 pathway using the drug omaveloxolone results in translocation of NRF2 to the nucleus, leading to increased transcription of SLC7A11 and other antioxidant response genes, increased GSH, and mitigation of the pathological phenotype of *Fdxr*^*R389W/R389W*^ mice and *FDXR* patient cells.

In this study, we provide evidence that loss of FDXR increases susceptibility to ferroptosis. The proposed molecular mechanism for this pathogenesis is summarized in Fig. 7. *FDXR* loss of function suppresses ISC biogenesis, which primarily impacts the role of multiple ISC-dependent complexes in the ETC. Mitochondrial iron accumulation results in an increase in ROS production from Haber-Weiss and Fenton reaction intermediates, causing an accumulation of lipid peroxidation in the IMM. Concurrently, insufficient OXPHOS increases reliance on glutamine metabolism to support anaplerotic processes, causing an influx of glutamine into the mitochondria, impacting the function of SLC7A11 (a necessary component of the cystine/glutamate antiporter system X_c_^-^), and causing cysteine deprivation and depletion of GSH [44]. With increasing IMM peroxidation and ROS accumulation, uncoupling of the mitochondrial membrane potential (MMP) occurs, emptying matrix components into the cytosol and introducing ROS and Fe^2+^ to the cytoplasm. With MMP destroyed, swelling and fragmentation of the mitochondrial network follows. Introduction of these matrix components, alongside GSH depletion, drives plasma membrane lipid peroxidation and eventual end-stage ferroptosis.

Given the multiple cellular pathways involved in this complex process, ferroptosis can be triggered by several different mechanisms including depletion of GSH through the system X_c_^−^/GSH pathway, inactivation of GPX4, depletion of GPX4 and CoQ10, or increasing the labile iron pool [16, 17]. Based on the results shown here, defective system X_c_^-^ and increased iron level appear to be important mechanisms underlying ferroptosis observed in *FDXR*^*R386W/R386W*^ mutant cells. In addition, ferroptosis and suppression of the NRF2 pathway are established pathologies in individuals with FRDA [92–94], which involves the disruption of another important ISC synthesis gene *frataxin* (*FXN*) with some functional similarities to FDXR. In both *FDXR*^*R386W/R386W*^ patient cells and the *Fdxr*^*R389W/R389W*^ mouse model, we similarly found depletion of not only NRF2, but of the NRF2-target and ferroptosis inhibitor SLC7A11. Loss of SLC7A11 would, in turn, cause cysteine deprivation and GSH depletion (i.e. the “class I” form of ferroptosis induction) [16, 17], contributing to increased membrane lipid peroxidation and cell death via ferroptosis (Fig. 7). This made the NRF2/SLC7A11 axis a particularly intriguing therapeutic target in the context of FDXR-related mitochondriopathy, given that it appears ideally suited to target the “class I” mechanism of ferroptosis.

NRF2 deficiency in FDXR-related disease provides a novel therapeutic target for treatment of this disease. The NRF2 activator, omaveloxolone, became a candidate drug to combat ferroptosis due to its ability to inhibit of KEAP1 ubiquitination. A recent clinical trial of omaveloxolone performed in FRDA patients reported improved neurological function assessment in the omaveloxolone-treated group relative to the placebo group after 48 weeks of treatment, with no major adverse events reported [81, 95]. Due to these clinical studies, omaveloxolone (now patented as Skyclarys®) was approved by the FDA for the treatment of FRDA in patients ages 16 years and older. Given that FXN- and FDXR-related diseases are both iron overload-ISC deficiency disorders, we hypothesized that omaveloxolone may also be an effective treatment for individuals with FDXR*-*related mitochondrial disease.

In support of this hypothesis, treatment of *FDXR*^*R386W/R386W*^ cells with omaveloxolone resulted in translocation of NRF2 to the nucleus, increasing expression of NRF2 target genes such as *SLC7A11*. Similarly, *Fdxr*^*R389W/R398W*^ mice showed increased expression of NRF2 and SLC7A11 following omaveloxolone treatment, as well as a corresponding improvement in survival and other morbidities such as paraphimosis (Fig. 6). Thus, as hypothesized, omaveloxolone treatment has anti-ferroptotic effects in *FDXR* loss-of-function cells by normalizing NRF2 and SLC7A11 levels, which in turn combats lipid peroxidation and blocks ferroptosis (Fig. 7). This is consistent with the previous literature showing that omaveloxolone acts by blocking the action of KEAP1 and subsequent NRF2 ubiquitination, leading to increased nuclear localization of NRF2 and activation of its downstream transcriptional targets [83]. However, further work will be needed to determine the mechanistic link between loss of FDXR and loss of NRF2 protein. Given that pathogenic variants in *FXN* have also been shown to cause decreased NRF2 protein [92–94], it is possible that the mechanism involves crosstalk between NRF2 and ISC biosynthesis [96].

The major finding of this study was the stabilization of ferroptosis features (such as lipid peroxidation and SCL7A11 expression), along with a corresponding phenotypic improvement in *Fdxr*^*R389W/R389W*^ mice treated with omaveloxolone. While significant, some caveats are required. FDXR-related mitochondriopathy likely encompasses multiple contributing pathologies. This presumably includes loss of the ability to synthesize important iron-sulfur cluster proteins, as well as adrenal insufficiency related to FDXR’s role in mitochondrial cytochrome P450 function [97], which we have recently shown to occur in both FDXR patients as well as the *Fdxr*^*R389W/R389W*^ mouse model [98]. If FDXR-related adrenal insufficiency is contributing significantly to the incidence of early death in some forms of FDXR related disease, this is likely to be unrelated to mitochondrial iron overload. In such cases, the benefits of upregulating NRF2/SLC7A11 and blocking ferroptosis may be limited, as the actual enzymatic functions of FDXR have not directly been restored. However, given the recent FDA approval of omaveloxolone for FRDA (another condition to which ferroptosis is a known pathology), our data showing normalization of ferroptotic features such as SLC7A11 supports exploration of this pharmaceutical for individuals affected by FDXR-related disease.

In summary, this study has shown that FDXR loss of function results in increased ferroptosis, likely originating from complex deficiencies and iron overload in the mitochondria. Treatment of *FDXR*^*R386W/R386W*^ cells and *Fdxr*^*R389W/R389W*^ mice with the NRF2 activator omaveloxolone decreased features associated with ferroptosis, suggesting omaveloxolone could be a possible treatment option for FDXR-related mitochondriopathy. Clinical trials to study the effectiveness of omaveloxolone to improve symptomology of FDXR-related mitochondriopathy, and perhaps other iron-related disorders, are indicated.

## Materials and methods

### Animal Models

Male and female mice from the C57BL/6 N background and between the ages of 0 and 130 days were utilized for the animal studies in this paper. Mice were housed in cages kept at consistent temperature, humidity and with a 12:12 light:dark cycle. Mice were fed standard chow (6.5% kcal fat, Tekland, 2020X) with free access to food and water. All animal research methods were reviewed and approved by the Cincinnati Children’s Hospital Medical Center (CCHMC) Institutional Animal Care and Use Committee and the University at Buffalo Institutional Animal Care and Use Committee.

The *Fdxr*^R389W/R389W^ transgenic mice were generated by the CCHMC Transgenic Animal and Genome Editing Core using previously described CRISPR/Cas9 gene-editing approaches [99, 100]. Briefly, donor constructs, sgRNA and Cas9 mRNA were injected into the cytoplasm of C57BL/6N zygotes. Seven pups were obtained and genotyped via PCR and sequencing. Since the donor construct to create the amino acid substitution also contained non-coding DNA substitutions that created a TspRI digest site, enzyme digests were also performed on the PCR products to identify potential candidates. Three of the mice were found to carry at least one copy of the intended knock-in allele, of which one (a female) was used to establish a stable genetic line. After establishing the mouse stock, *Fdxr*^*R389W/R389W*^ mice were generated by crossing heterozygous *Fdxr*^*R389W/+*^ mice. DNA for genotyping was collected using tail or ear snips. The genotypes were determined through PCR using the following primers: *Fdxr* genotyping forward (GCAGCGTTGGGTATAAGAGC) and *Fdxr* genotyping reverse (ACTGATGCTCCCCAAGGAC). The genotypes were then confirmed either with Sanger sequencing or via TspRI digest of the PCR products.

### Phenotypic measurements and behavioral assays in mice

*Fdxr*^*R389W/R389W*^ mice and *Fdxr*^*+/+*^ littermates were observed for survival rates. Weight was measured weekly in these cohorts. To assess neurological function, a group of age- and sex-matched *Fdxr*^*R389W/R389W*^ and *Fdxr*^*+/+*^ littermates, were selected to undergo behavioral testing. Behavioral tests were performed at the CCHMC Animal Behavioral Core by trained laboratory technicians, using previously described protocols [67, 101]. Locomotor activity was determined through an automated behavioral assay which recorded parameters such as horizontal activity, vertical movement, total distance traveled (cm), and repetitive movements. Additionally, the San Diego Instruments SR Apparatus was used to measure tactile startle response (TSR), acoustic startle response (ASR) and pre-pulse inhibition (PPI) to assess autonomic function via reflex and startle reactions.

### Mitochondrial respiratory chain complex I–IV activity assay

Mitochondrial complex activities of complexes I, II, III, and IV were assayed from mouse mitochondrial heart extracts, as previously described [67]. Briefly, mitochondrial complex I activity was determined by measuring the descending NADH absorbance at 340 nm, with decylubiquinone used as an electron acceptor. Complex II and III activity levels were assessed by the inclining absorbance of reduced cytochrome C at 550 nm; potassium cyanide was utilized in the medium to inhibit the subsequent oxidation. Complex IV activity was determined using the Complex IV Rodent Enzyme Activity Microplate Assay Kit (Abcam, United Kingdom). The activity of complex IV was measured by testing the oxidation of reduced cytochrome c, which was determined by monitoring the dynamic of absorbance at 550 nm. All absorbance values of the mitochondrial complex assays were recorded using a Synergy H1 microplate reader (Biotek Instruments, VT, USA). The relative enzymatic activity was calculated as nmol/min/mg protein and normalized to the corresponding wildtype activity levels.

### EMG recordings

Electromyograph (EMG) recordings were collected as previously described [67, 100]. Briefly, mice were first anesthetized with isoflurane inhalation, followed by 50 mg/kg sodium pentobarbital intraperitoneal injection. Next, the lateral gastrocnemius muscle of the mice was exposed from the knee to approximately 4 mm above the ankle, and the sciatic nerve was exposed near the biceps femoris. We implanted a polyester film wrap line on the outside of the mouse gastrocnemius and inserted a reference line below the skin near the tail of the mouse. We placed a concentric bipolar stimulation electrode on the sciatic nerve of the mouse for electrical activation. The composite muscle action potential (CMAP) was amplified and acquired using a Micro 1401 Data Acquisition Unit and analyzed offline using Spike2 software (Cambridge Electronic Design, Cambridge, UK). The sciatic nerve adjacent to the tibia and fibula received 2–4 mA electrical stimulation through a stimulus isolator (World Precision Instruments, Sarasota, FL) attached to a Micro 1401 data acquisition unit. After the data recording was complete, the sciatic nerve was axotomized, and the distal sciatic nerve was stimulated to confirm that direct neural stimulation still produced CMAP, while the proximal sciatic nerve did not. We calculated conduction velocity (CV) by calculating the latency between stimulation and the recorded response, divided by the length of the sciatic nerve from stimulation site to recording site for each animal.

### Electron microscopy

Mice were euthanized in age-matched groups and perfused with 0.9% saline. Tissues were fixed in 10% formalin, then embedded in paraffin and sectioned. Selected sections were stained with hematoxylin and eosin (H&E). H&E-stained tissues were analyzed by light microscopy. Images were obtained under light microscopy (BX63; Olympus Corporation; Center Valley, PA). Select sections were then counterstained with uranyl acetate 2% (EMS) and lead citrate (EMS) and examined by TEM. All TEM images were taken with an 80-kV transmission electron microscope (Hitachi, H-7650, V01.07, Tokyo, Japan). G-ratio was then calculated as the ratio of the inner-to-outer diameter of a myelinated axon, which were measured from TEM images using ImageJ software.

### Cell Lines

All participants who offered biopsy samples for the fibroblast cell line described in this study provided written and informed consent to participate in this research. Written consent was obtained and archived for all participants or their legal guardians. All human subjects research related to this paper received Institutional Review Board (IRB) approval consistent with the principles of research ethics and the legal requirements of the lead author’s jurisdiction(s), through the State University at Buffalo (FWA00008824). Skin punch biopsies from patients with biallalic, pathogenic *FDXR* variants were used to generate the fibroblast lines from study participants using the procedure described below. Briefly, a skin biopsy (3-mm diameter) was collected from the upper shoulder of consented FDXR-related mitochondriopathy participants, dissected into small pieces, incubated in 0.7% collagenase (type B) for 1.5-2.5 h at 37° C, and plated into 1 to 2 T25 flasks containing AmnioMAX™ C-100 Complete Medium (Thermo Fisher Scientific, Waltham, MA, USA). Fibroblast cultures were subsequently maintained in Dulbecco’s Modified Eagle Medium (DMEM) (Thermo Fisher Scientific), supplemented with 10% Fetal Bovine Serum (FBS) (Thermo Fisher Scientific). Patient-derived fibroblasts were periodically tested for mycoplasma contamination using the MycoAlert Mycoplasma Detection Kit (Lonza Biosciences, Walkersville, Maryland, USA). Their identity was also periodically authenticated by testing for the presence of their expected biallelic *FDXR* variants using Sanger sequencing. A primary normal human dermal fibroblasts (NHDF) cell line was used as a control for all functional studies reported in this manuscript. The cell line was obtained from a commercial vendor (ATCC, Manassas, Virginia, USA), who perform quality control testing, authentication and mycoplasma testing prior to shipping.

### Reverse transcriptase PCR (qRT-PCR)

Total cellular RNA was isolated from tissues using E.Z.N.A. Total RNA Kit I (Omega Bio-Tek), according to the manufacturer’s instructions. qRT-PCR reactions were set up using the Brilliant III Ultra-Fast SYBR Green QRT-PCR Master Mix (Agilent), according to the manufacturer’s instructions. Results were quantified according to the 2^-dCt method, using *Tubb2a* (*beta-tubulin*) as the reference gene for mice samples and *beta-actin* as the reference gene for human samples. The following primers were used to recognize the genes of interest: Mouse Fdxr forward (GCAGCGTTGGGTATAAGAGC), Mouse Fdxr RT-PCR reverse (CTGGTGAGGAAGCTGTCTGTC), Mouse Tubb2a RT-PCR forward (TCCACCCCTTCTACAACCAG), Mouse Tubb2a RT-PCR reverse (TCCAGCTGCAAGTCACTGTC), Human *FTH1* forward (CCATCAACCGCCAGATCAAC), Human *FTH1* reverse (GCCACATCATCTCGGTCAAA), Human *FTMT* forward (AGCACATCAGCTCTGCACTG), Human *FTMT* reverse (AGGCCAGTAGGGGACCTAAA), Human *HMOX1* forward (GGTGATGGCTTCCTTGTACC), Human *HMOX1* reverse (AGTGAGGCCCATACCAGAAG), Human *SLC40A1* forward (GTGGAGTACTTCTTGCTCTGG), Human *SLC40A1* reverse (CTGCTTCAGTTCTGACTCCTC), Human *TFRC* forward (CTCAGTTTCCGCCATCTCAGT), Human *TFRC* reverse (GCAGCTCTTGAGATTGTTTGCA), Human *GCLM* forward (GCCACCAGATTTGACTGCCTTT) Human *GCLM* reverse (CAGGGATGCTTTCTTGAAGAGCTT), Human *PGD*forward (AGAGGCTTGGCCCCACAT),Human *PGD*reverse (CCGGTTCCCACTTTTGCA), Human *SLC7A11* forward (GACGATGGTGATGCTCTTCTC), Human *SLC7A11* reverse (TGGGCGTTTGTATCGAAGATA), Human *TXNRD1* forward (AAAGACGATGAACGTGTCG), Human *TXNRD1* reverse (CTTAGTCAGCCCACACTTGAG), Human *beta-actin* forward (CCAGCCTTCCTTCTTGGGTAT), Human *beta-actin* reverse (GGGTGTAAAACGCAGCTCAG).

### Western blot

Western blot analysis was performed as previously described [102]. For isolation of nuclear and cytoplasmic extract from fibroblasts, the Cayman Chemical Nuclear Extraction Kit (Catalog #10009277) was used according to the manufacturer’s protocol. For omaveloxolone treatment, fibroblast cells were cultured with 50 nM omaveloxolone solution or DMSO solution and incubated for 72 h prior to protein collection. The following primary antibody dilutions were applied: rabbit anti-NRF2 (16396-1-AP, Proteintech) at 1:1000 dilution, rabbit anti-FDXR (ab204310, Abcam) at 1:1000 dilution. For staining SLC7A11 protein, rabbit anti-SLC7A11 (PA1-16893 Thermo Fisher) was used at 1:1000 dilution for staining mouse tissues, and rabbit anti-SLC7A11 (12691, Cell Signaling Technology) at 1:1000 for staining human cells. For myelin basic protein (MBP), antibody was obtained from Proteintech (10458-1-AP) and used at 1:1000 dilution. DELE1 antibody was obtained from Santa Cruz (sc-515080). For loading controls, the following primary antibodies were used, depending on the experiment: mouse anti-beta-actin antibody (NP600-501, Novus Biologicals) at 1:2000 dilution, mouse anti-Lamin A/C (4777, Cell Signaling Technology) at 1:1000 dilution, or mouse anti-GAPDH (CB1001, Sigma-Aldrich) at 1:2000 dilution. Secondary antibodies and dilutions are as follows: goat anti-Rabbit HRP (A16104, Invitrogen) at 1:5,000 dilution, or goat anti-mouse HRP (31432, Invitrogen) at 1:5,000 dilution. Full and uncropped images for all western blot images in this manuscript can be found in file title “Supplemental Material – Uncropped Western Blots”).

### Immunohistochemistry

For staining of mouse cerebellum, cryosections were first collected as previously described [100, 103]. Briefly, mice were perfused transcardially with PBS and fresh 4% paraformaldehyde (PFA) in PBS. After overnight postfixation in 4% PFA at 4 °C, tissues were washed twice in 30% sucrose and then once more overnight in 30% sucrose. After the overnight sucrose treatment, tissues were frozen in OCT, and the resulting blocks were sectioned into 12-16 micron cryosections and collected onto slides for IHC staining. For GFAP staining, sections were stained in the CCHMC pathology core with rabbit anti-GFAP (Cell Signaling Technology, #12389) at 1:1000 dilution. For Fluoro-Jade C staining, staining was performed using the “Fluoro-Jade C (FJC) Ready-to-Dilute Staining Kit for identifying Degenerating Neurons” (Biosensis), according to the manufacturer’s instructions. Images were collected on either a Zeiss Axioskop microscope (for color images) or an Andor Dragonfly Spinning Disk Confocal Microscope (for fluorescent images).

For live-cell imaging, participant-derived and control fibroblasts were seeded at a density of 5 ×10^4^ on 35 mm glass bottom dishes and cultured for 48 h. For omaveloxolone treatment, this 48 incubation period also included 50 nM omaveloxolone (RTA-408) to activate the NRF2 pathway, or vehicle-only control (DMSO). Following this, erastin (5μm) or DMSO control was added to cell culture and incubated for 15 h to induce ferroptosis. After induction, MitoCLox (200 nM), Mito-FerroGreen (5 μM), or BODIPY-C11 581/591 stain (1.5 μM) was added to cell culture, with the addition of Hoechst 33342 staining to mark the nucleus (1: 2000). After the recommended incubation time, cells were washed twice with PBS and imaged using an Andor Dragonfly Spinning Disk Confocal Microscope.

### Cell viability assay

NHDF and FDXR mutant cells were plated on 96-well plates at a density of 4 × 10^3^ cells in 100 μl per well. For erastin treatment, cells were treated with erastin (5 μM) or vehicle (DMSO) for 15 h, followed by cell viability assay using CyQUAN XTT Cell Viability assay kit (Invitrogen, X12223). The viability of NHDF treated with vehicle was set to 1. For RTA-408 treatment, cells were treated with RTA-408 (50 nM) or vehicle (DMSO) for 48 h, followed by erastin (5 μM) treatment and viability assay. Iron chelator deferoxamine (DFO) was purchased from ApexBio Technology (Catalog No. B6068).

### Detection of ferroptosis via flow cytometry

Detection of ferroptosis via Flow cytometry was performed as previously described [104]. Briefly, participant and control fibroblasts were seeded at a concentration of 1 × 10^6^ cells per 6 well plate and incubated for 48 h to allow for attachment. After incubation, erastin (5 μM) or DMSO control was added to cell culture to induce ferroptosis and incubated for 15 h. After erastin treatment, MitoCLox (200 nM), Mito-FerroGreen (5 μM), or BODIPY-C11 581/591 stain (1.5 μM) was added to cell culture, in addition to Hoechst 33342 staining to mark the nucleus of cells (1: 2000). Cells were trypsinized, washed twice with PBS + 0.5% FBS, 2 mM EDTA and analyzed using a BD LSRFortessa Cell Analyzer. The PE-Texas Red channel was used to quantify red fluorescence and Alexis-Flour 488 or FITC channel was used to measure green fluorescence. MitoTracker Red and MitoTracker Green were used as single-color positive controls. Gating strategy for analysis of cells is demonstrated in Supplementary Fig. S7. Doublet cells were gated out, following by exclusion of cell debris. As we aimed to capture both living and dead cells undergoing ferroptosis, Hoechst 33342 staining was selected as it marks the nucleus of both living and dead cells. Data was analyzed using FCSalyzer software.

### Omaveloxolone treatment for *Fdxr*^*R389W/R389W*^ and *Fdxr*^*+/+*^ mice

Shortly after birth, *Fdxr*^*R389W/R389W*^ and *Fdxr*^*+/+*^ mice were selected and placed in treatment or vehicle control cohorts. Mice were administered omaveloxolone (RTA-408, Cat. No. HY-12212, MedChemExpress) at a dosage of 17.5 mg per kg mouse bodyweight or vehicle alone (0.5% DMSO and 0.5% TWEEN-20) via daily intraperitoneal injections. Treatment was initiated at 3 weeks of life and continued until end of life, or until the mouse was sacrificed for tissue extraction. For assessment of overall survival rates, cohorts of *Fdxr*^*R389W/R389W*^ and *Fdxr*^*+/+*^ mice, treated with omaveloxolone or DMSO vehicle control, were followed until end of life.

### Statistical analysis

For experiments performed for this paper, the sample size for each experimental group/condition is provided where relevant in each figure legend. Sample sizes were chosen based on prior experimental experience in studying mitochondrial disease models in animal and cell culture. For animal behavioral studies, the personnel performing the assays were blinded to the experimental groups during performance of the assays and collection of the data. The genotypes of the experimental groups were unblended for subsequent statistical analysis. No randomization method was used for assignment of animals to experimental group. For the calculation of g-ratio from optic nerve fibers, the relative diameter of the total fiber and the inner axon were manually determined from transmission electron micrographs using ImageJ software.

Statistical analysis was carried out using GraphPad Prism software. Unless indicated otherwise, a p-value < 0.05 was considered statistically significant. For each dataset, an estimation of variation was made within each group of data, and an F-test to compare variance between groups was performed to determine if the relevant groups had statistically different variance. Normality testing was also performed for each group of data using the Shapiro-Wilk test to determine if the data is normally distributed. For any data that failed either the normality test or the equal variance testing, nonparametric statistical tests were used. The specific parametric or nonparametric tests used are defined in the relevant figure legends.

## Supplementary information

Supplemental Materials - Supplementary Fig.s

Supplemental Materials - Uncropped Western Blots

## Data Availability

De-identified data and materials are available upon request.
