# Supplementary material for: Ferroptosis is a novel pathogenic mechanism of FDXR-related disease via disruption of the NRF2 pathway
Source: Cell Death Discov. 2025 Dec 23;11:563. doi: 10.1038/s41420-025-02840-y (PMC12727864; doi:10.1038/s41420-025-02840-y)
Supplement: Supplementary file 1 — Supplemental Materials - Supplementary Fig.s [file 41420_2025_2840_MOESM1_ESM.pdf]

SUPPLEMENTAL MATERIAL

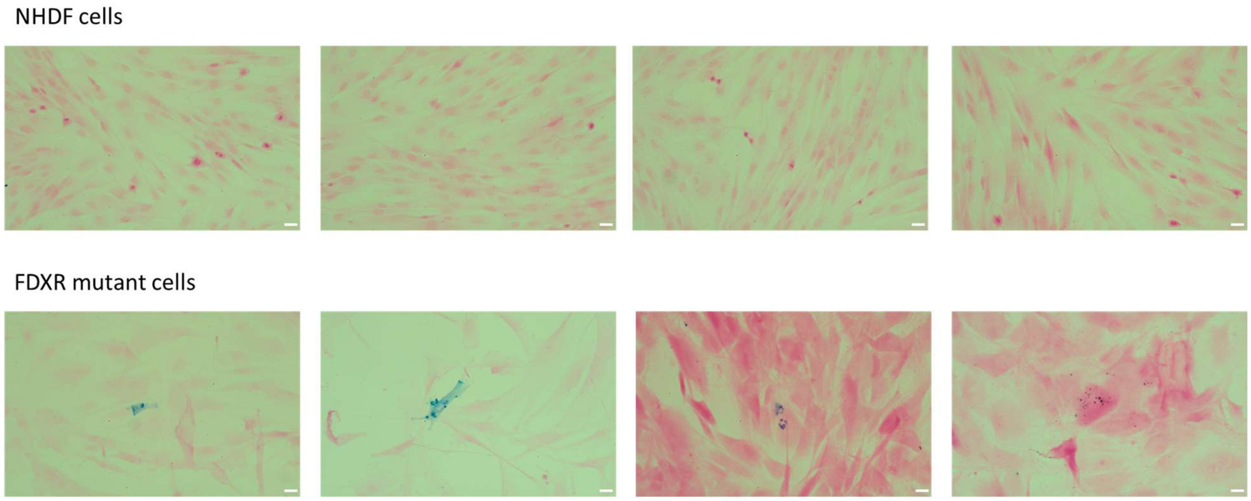

Fig. S1. Iron accumulation in *FDXR*$^{R386W/R386W}$ cells as compared to NHDF cells. NHDF and *FDXR*$^{R386W/R386W}$ cells were plated on 35mm glass bottom dishes at density of $5x10^4$ cells in 200 µl and cultured for 24 hours. Prussian blue staining was performed using the iron staining kit from Sigma (HT20-1KT). Images were taken using an AxioSkop microscopy. Scale bar: 25 µm.

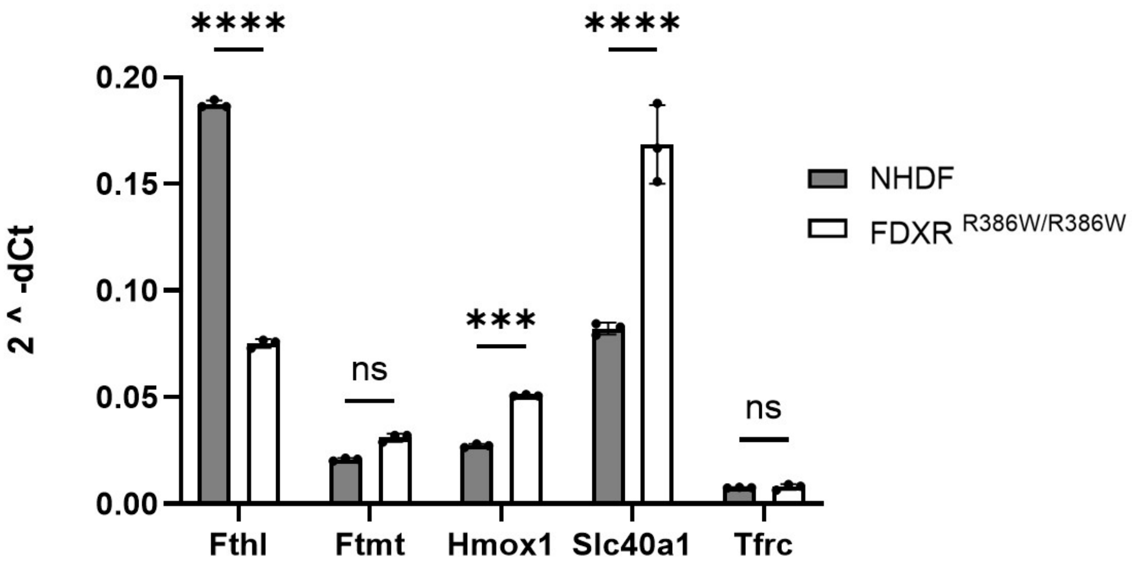

Fig. S2. Quantitative RT-PCR analysis reveals altered expression of iron-metabolism related genes in *FDXR*$^{R386W/R386W}$ cells as compared to NHDF cells. Fthl, ferritin heavy chain 1; Ftmt, mitochondrial ferritin; Hmox1, heme oxygenase 1; Slc40a1, ferroportin 1; Tfrc, transferrin receptor. Statistical testing was performed using a Two-way ANOVA omnibus test, followed by post hoc testing using Šídák's multiple comparisons test to determine individual p-values. Results are presented as means ± SEM.; ns=not significant, ***$p < 0.001$, ****$p < 0.0001$.

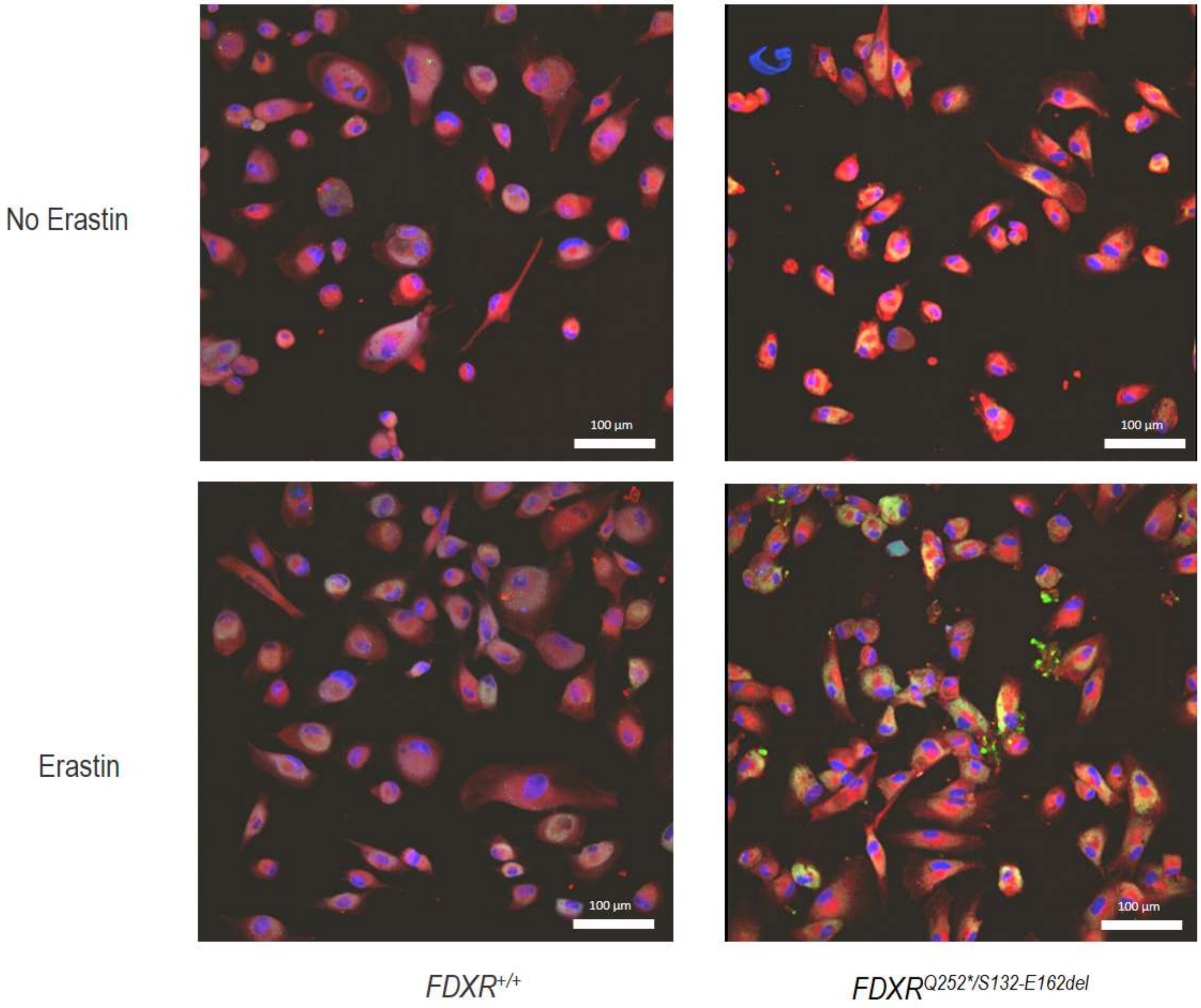

Fig. S3. Erastin induced ferroptosis in varying participant-derived fibroblasts. FDXR+/+, NHDF fibroblasts; *FDXR*$^{Q252*/S132\text{-}E162del}$, Fibroblasts from FDXR Study Patient 14 carrying a Q252* mutation and an S132-E162del splice mutation. Scale bar: 100 µm.

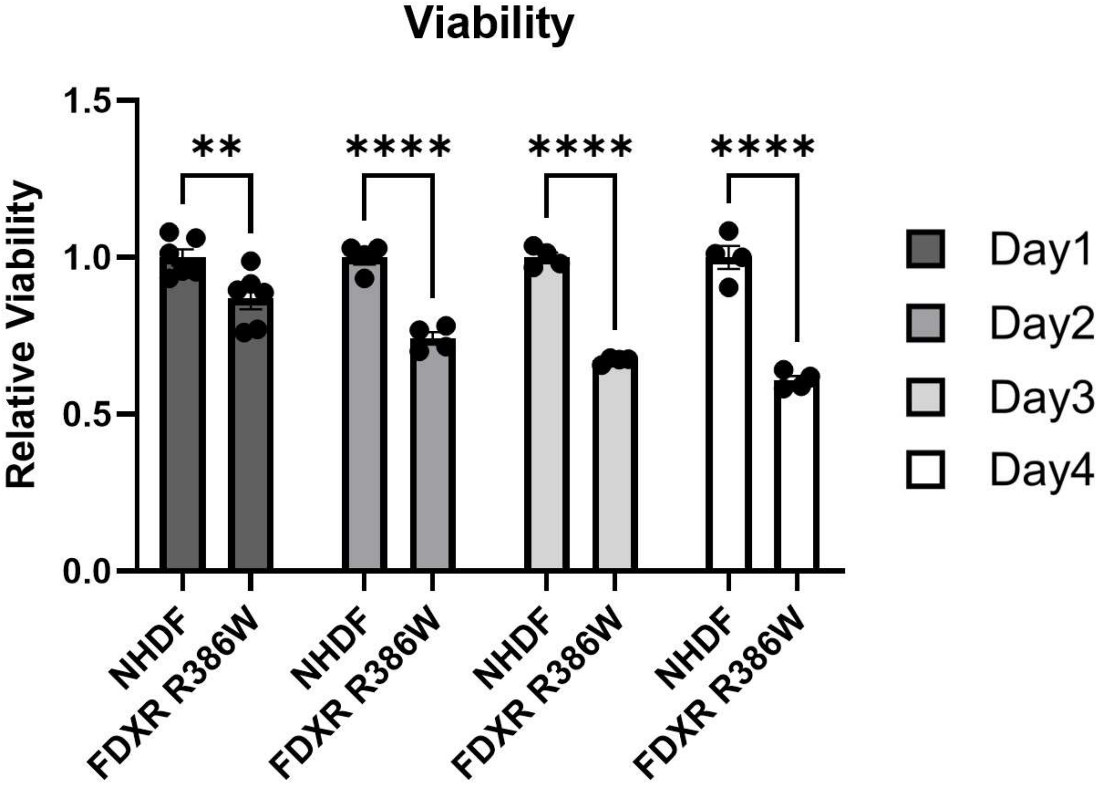

Fig. S4. Cell viability was reduced in *FDXR*$^{R386W/R386W}$ cells as compared to NHDF cells. NHDF and *FDXR*$^{R386W/R386W}$ cells were plated on four 96 well plates at density of 4x10^3 cells in 100 µl per well. Cell viability assay was performed everyday afterward for 4 days using CyQUAN XTT Cell Viability assay kit (Invitrogen, X12223). Statistical testing was performed using a Two-way ANOVA omnibus test, followed by post hoc testing using Šídák's multiple comparisons test to determine individual p-values. Results are presented as means ± SEM.; ns=not significant, **$p < 0.01$, ****$p < 0.0001$.

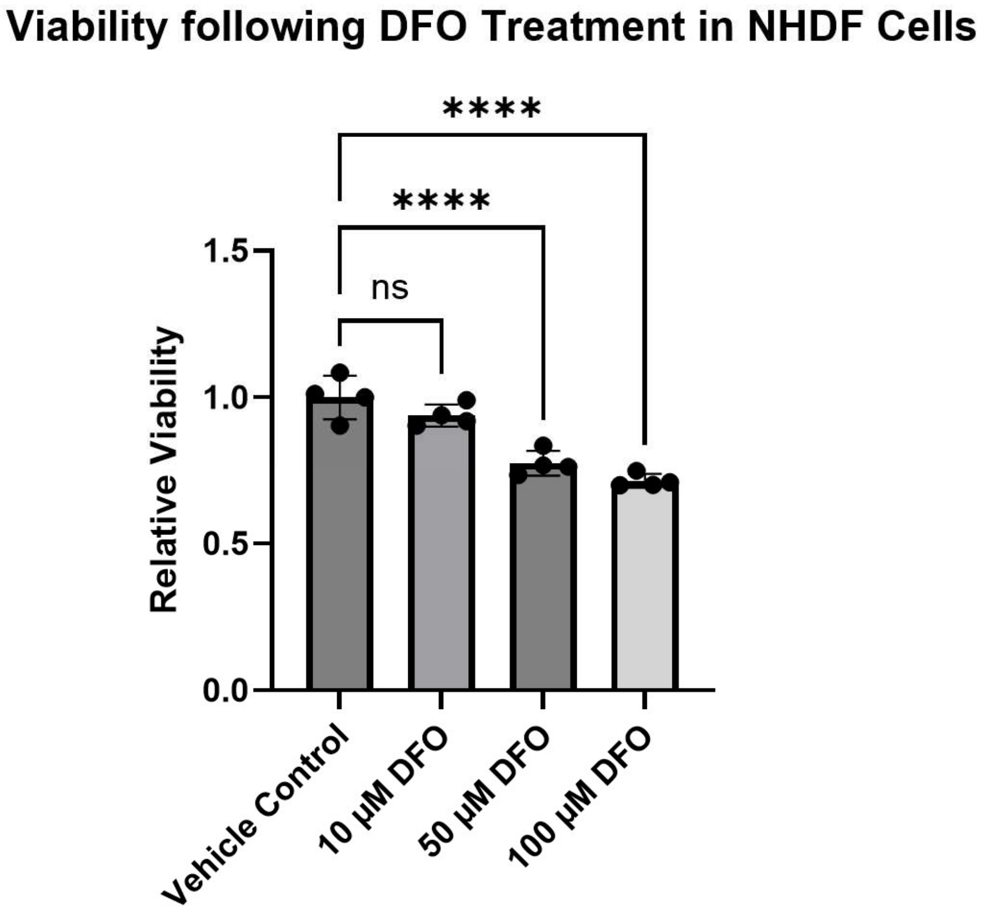

Fig. S5. DFO reduces viability of NHDF cells. NHDF cells were plated on a 96 well plate at density of $4x10^3$ cells in 100 µl per well and treated with DFO at concentrations of 0, 10, 50, and 100 µM. Three days after treatments, cell viability assays were performed. Statistical testing was performed using a One-way ANOVA omnibus test, followed by post hoc testing using Dunnett's multiple comparisons test to determine individual p-values. Results are presented as means ± SEM.; ns=not significant, ****$p < 0.0001$.

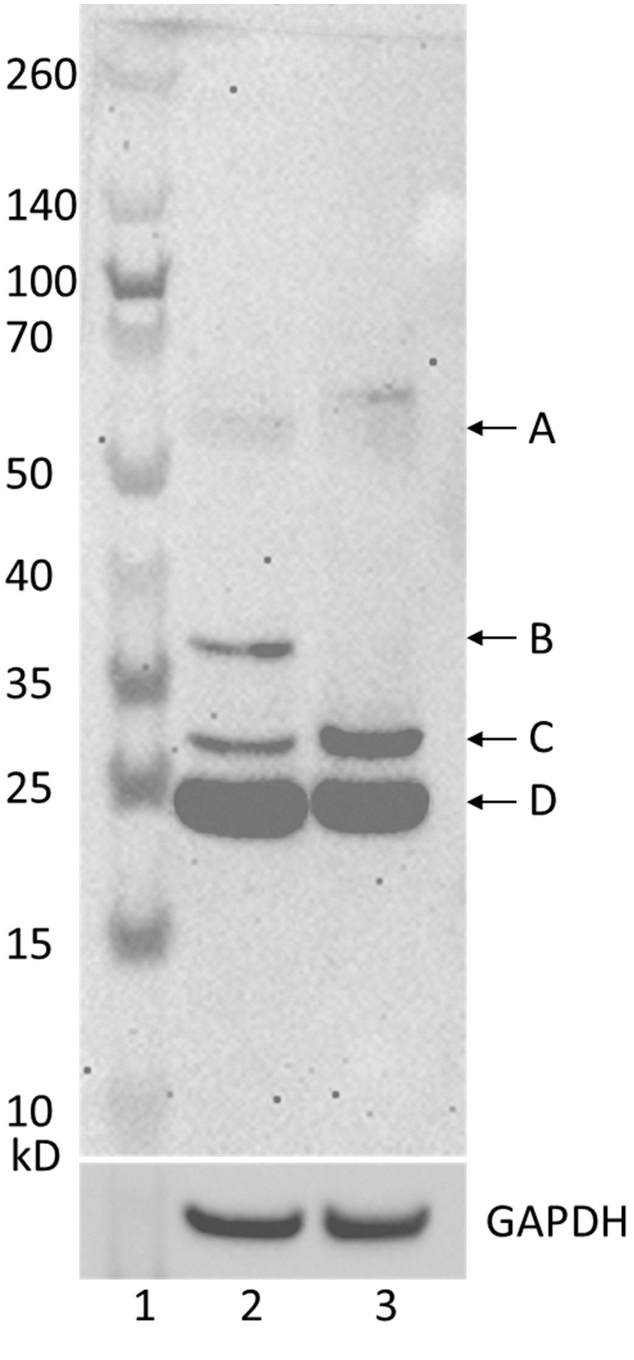

Fig. S6. Detection of DELE1 protein in NHDF and *FDXR*$^{R386W/R386W}$ cells. Whole cell lysates from NHDF and *FDXR*$^{R386W/R386W}$ cells were used for western blot using DELE1 antibody (sc-515080). Four bands (A, B, C, and D) were seen in NHDF cells. The approximate size of bands A, B, C, and D are 56, 37, 29, and 24 kD, respectively. Band B was undetectable in FDXR mutant cells. Band C was markedly increased in FDXR mutant cells. GAPDH was probed as a loading control. Lane 1: Protein ladder (Thermo Scientific #26634). Lane 2: NHDF. Lane 3: FDXR mutant.

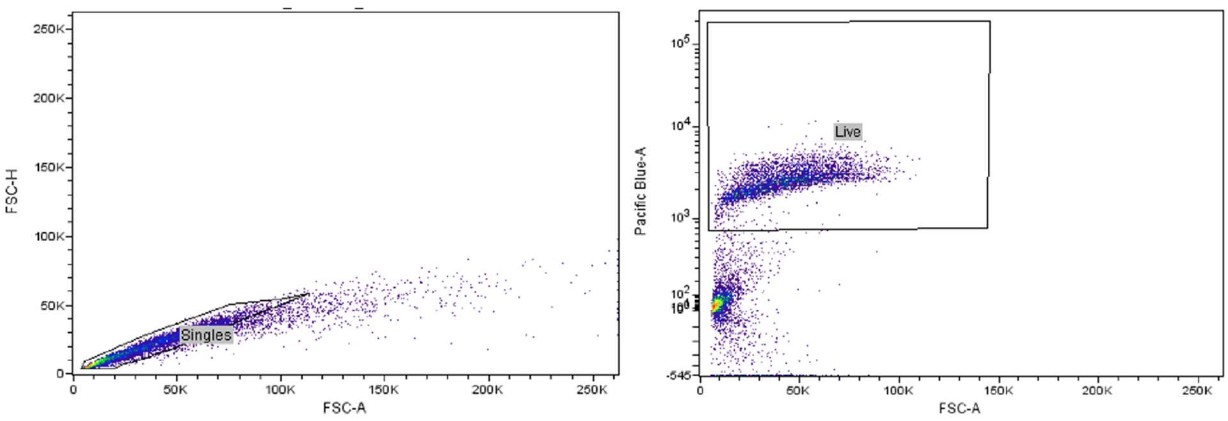

Fig. S7. Gating strategy for detection of ferroptosis via Flo cytometry. Pacific Blue, Hoechst 33342 staining.
