# Supplementary material for: Ferroptosis is a novel pathogenic mechanism of FDXR-related disease via disruption of the NRF2 pathway
Source: Cell Death Discov. 2025 Dec 23;11:563. doi: 10.1038/s41420-025-02840-y (PMC12727864; doi:10.1038/s41420-025-02840-y)

Figure 1B

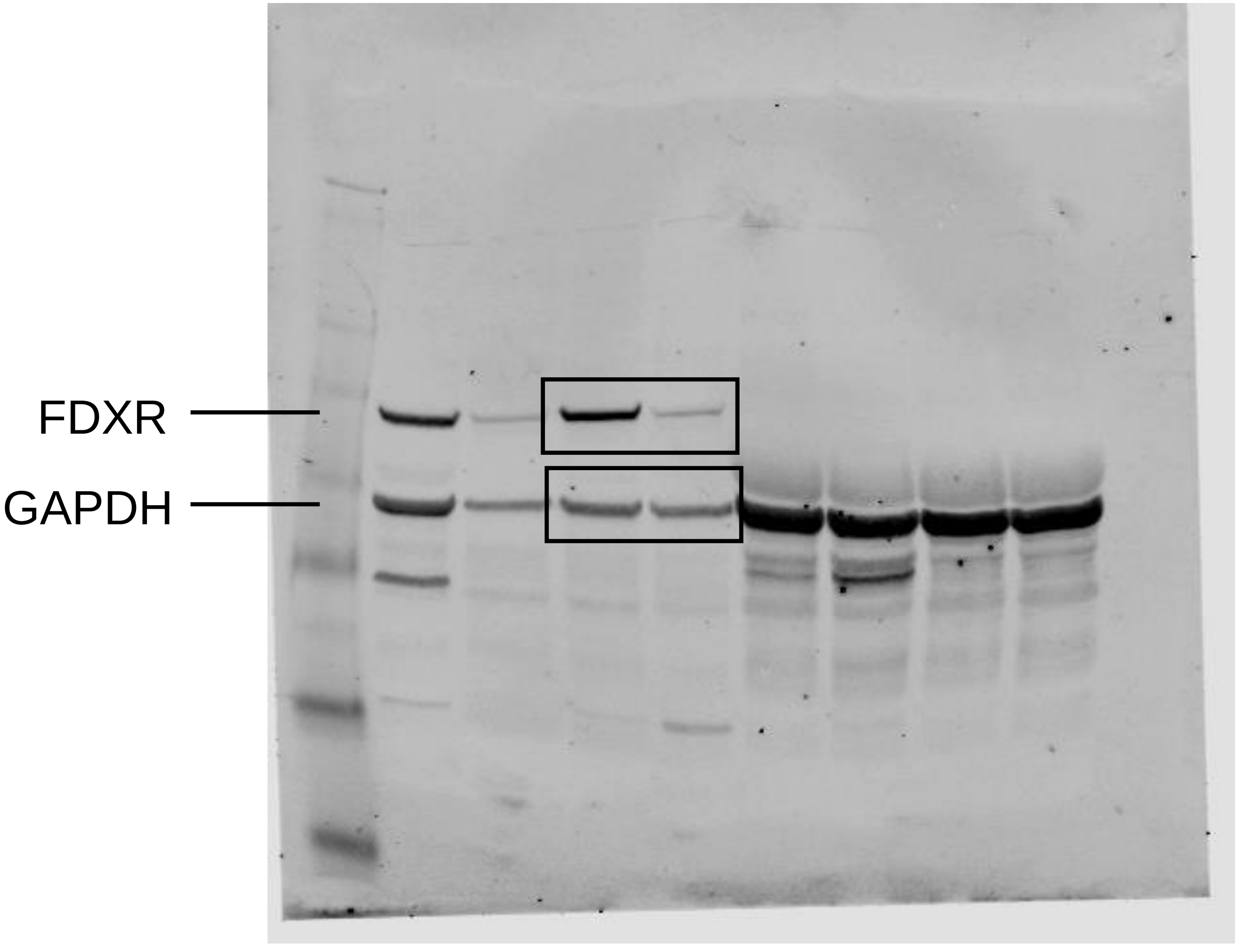

Figure 2F

MBP

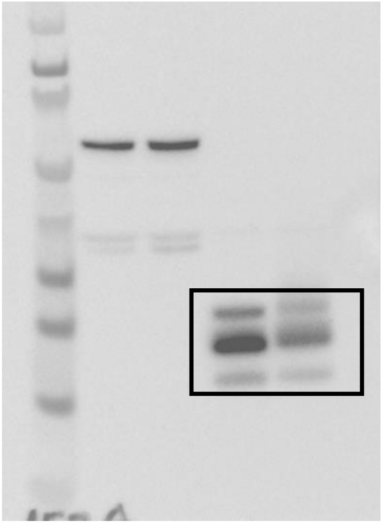

Actin

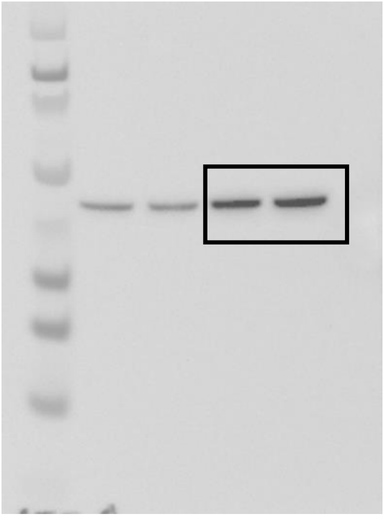

Figure 4A

NRF2

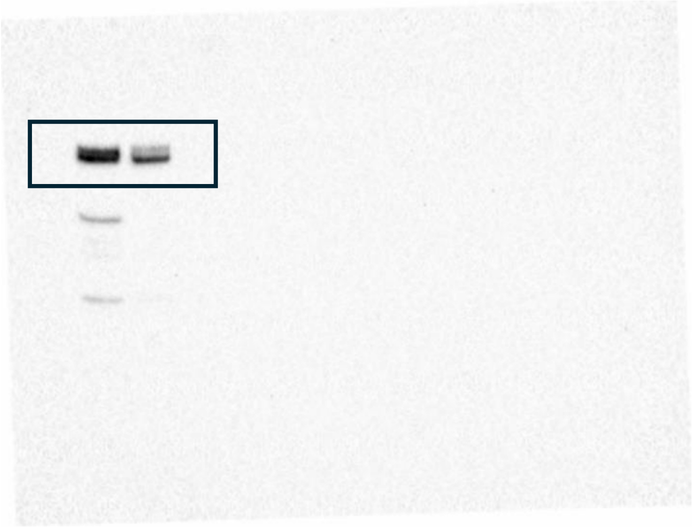

GAPDH

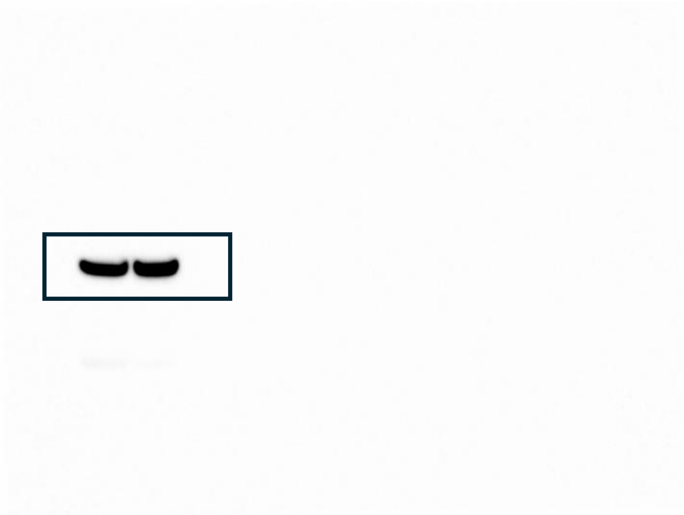

Figure 4B

NRF2

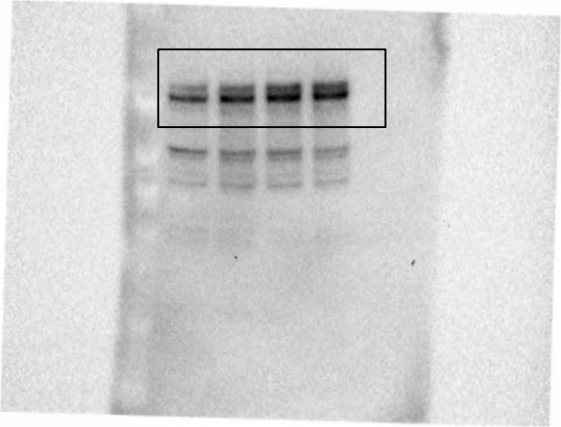

Actin

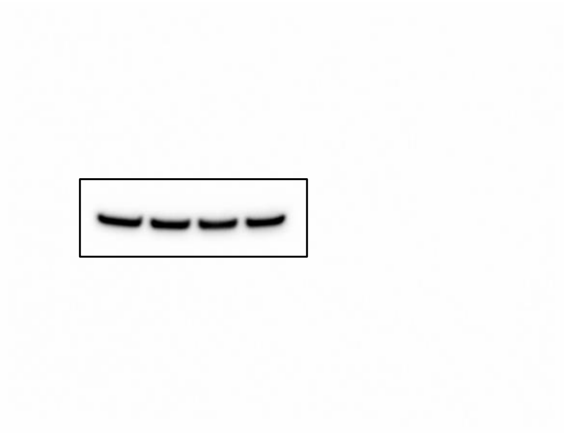

Figure 4C

Slc7a11

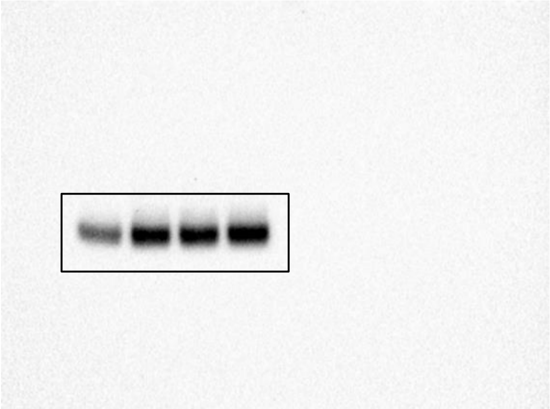

Actin

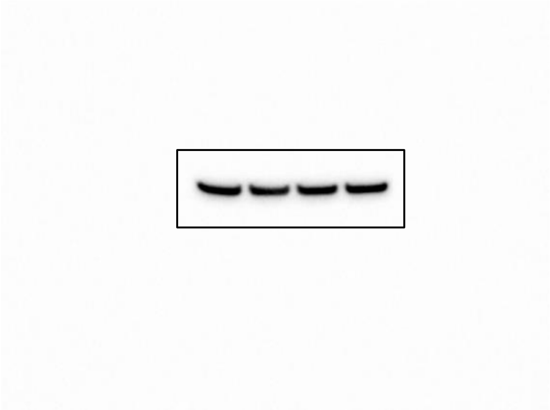

Figure 4D

NRF2

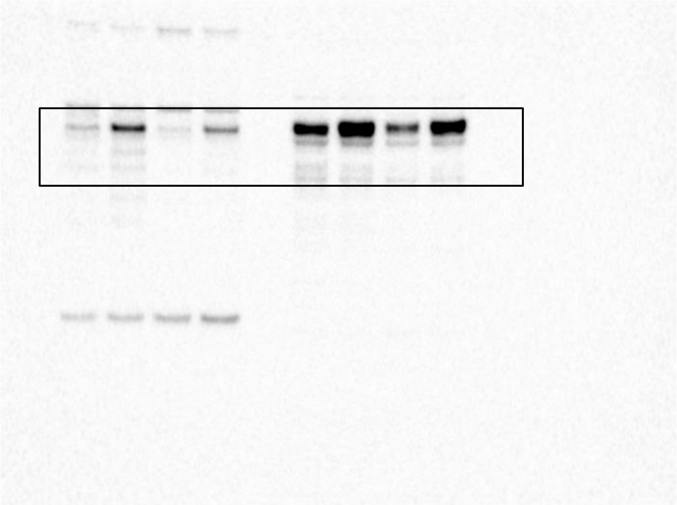

Lamin A/C

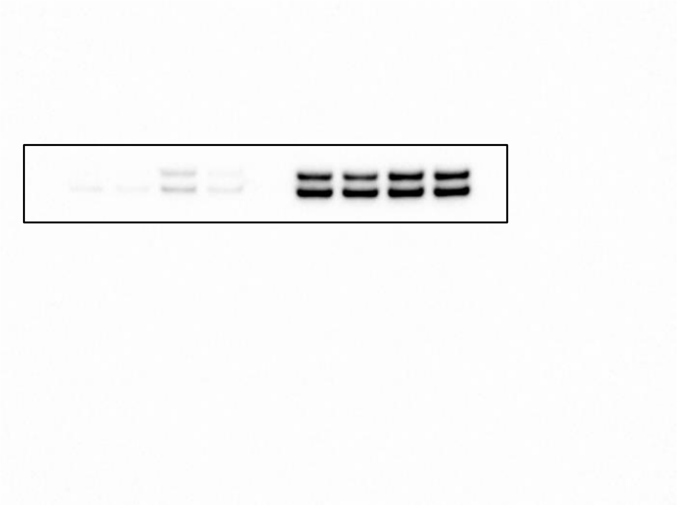

Figure 4D (Continued)

GAPDH

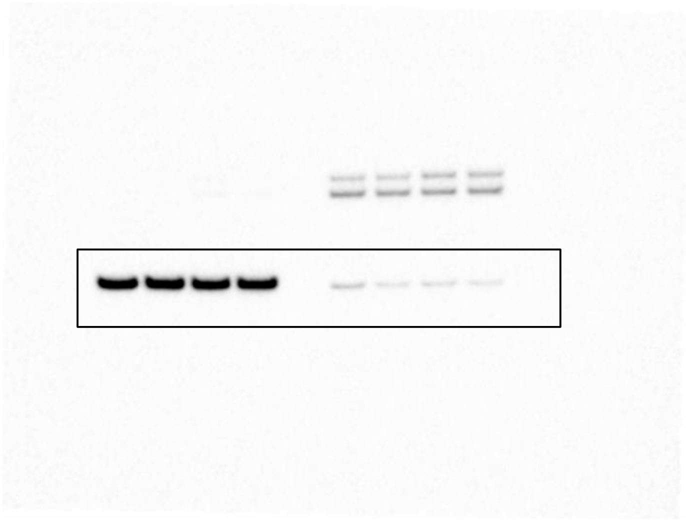

Figure 4F

Slc7a11

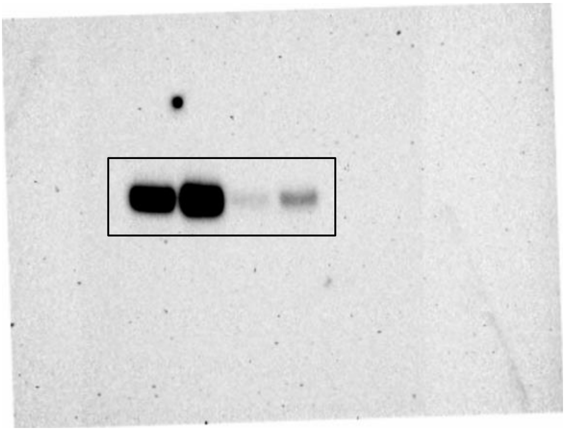

Actin

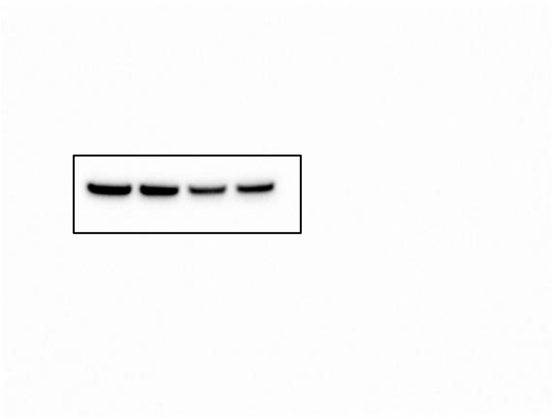

Figure 6E

Slc7a11

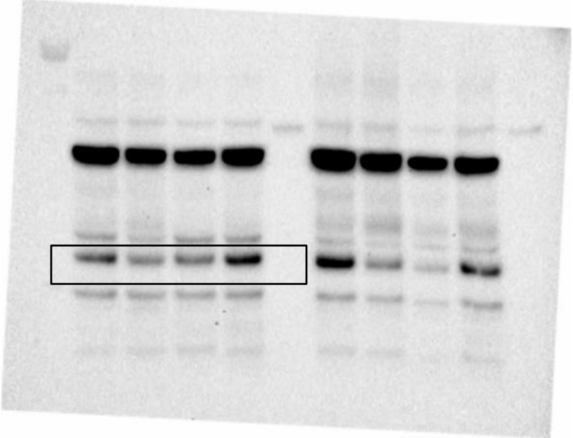

Actin

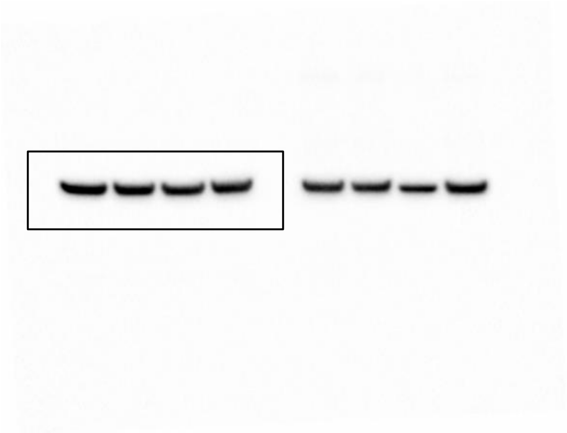

Figure 6E (Continued)

Nrf2

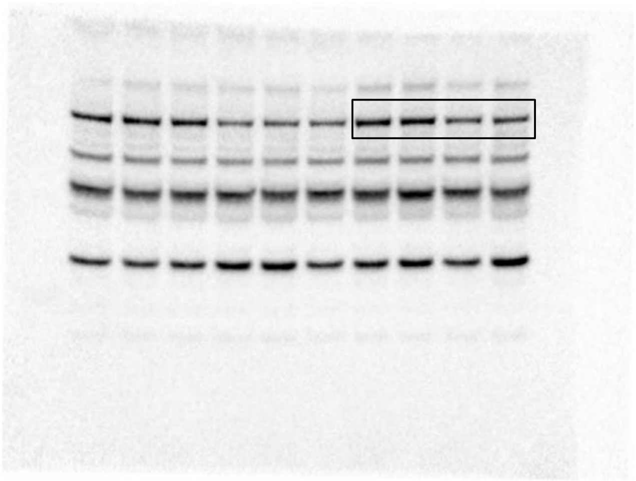

Actin

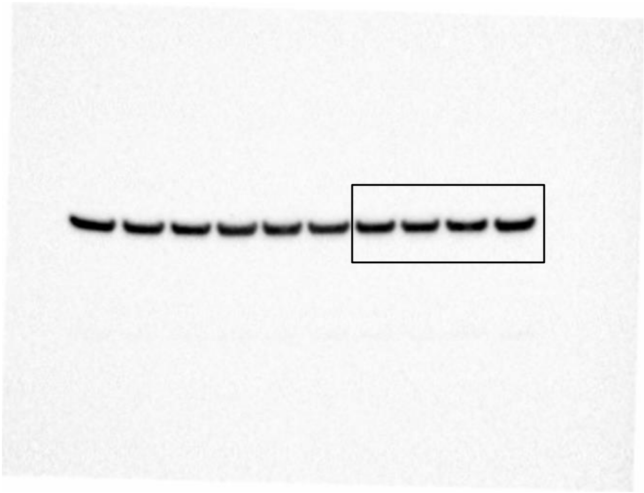

Supplemental Figure S7

DELE1

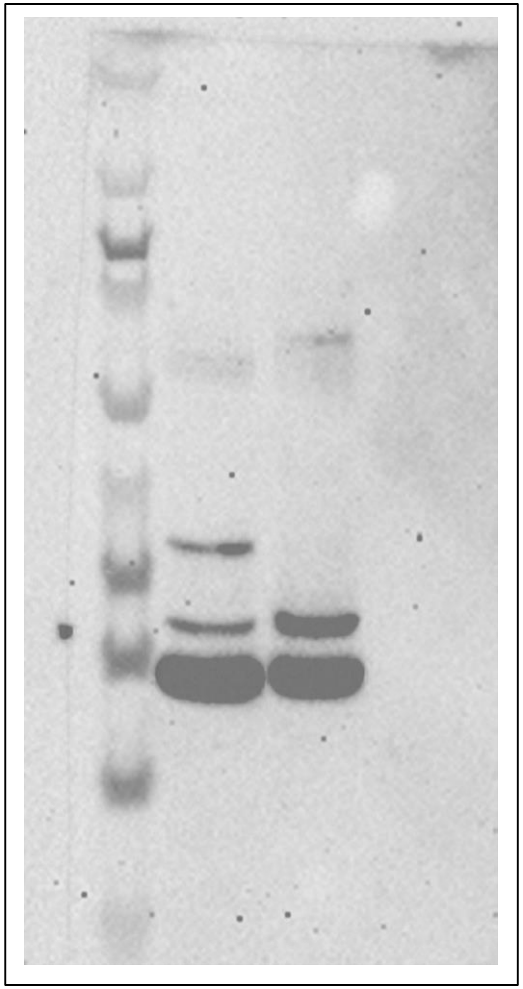

GAPDH

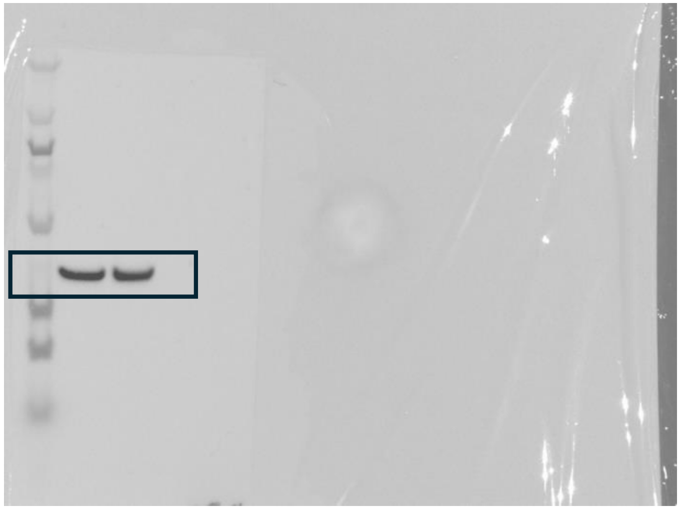

Supplement: Supplementary file 2 — Supplemental Materials - Uncropped Western Blots [file 41420_2025_2840_MOESM2_ESM.pdf]
